# Metabolomic profiling reveals severe skeletal muscle group-specific perturbations of metabolism in aged FBN rats

**DOI:** 10.1007/s10522-014-9492-5

**Published:** 2014-03-21

**Authors:** Sean M. Garvey, Janis E. Dugle, Adam D. Kennedy, Jonathan E. McDunn, William Kline, Lining Guo, Denis C. Guttridge, Suzette L. Pereira, Neile K. Edens

**Affiliations:** 1 Abbott Nutrition R&D, 3300 Stelzer Road, Bldg RP4-2, Columbus, OH 43219 USA; 2 Metabolon, Inc., 617 Davis Drive, Suite 400, Durham, NC 27713 USA; 3 Human Cancer Genetics Program, The Ohio State University, 460 West 12th Avenue, BRT910, Columbus, OH 43210 USA

**Keywords:** Muscle, Aging, Metabolomics, Sarcopenia, Biomarkers, NAD

## Abstract

Mammalian skeletal muscles exhibit age-related adaptive and pathological remodeling. Several muscles in particular undergo progressive atrophy and degeneration beyond median lifespan. To better understand myocellular responses to aging, we used semi-quantitative global metabolomic profiling to characterize trends in metabolic changes between 15-month-old adult and 32-month-old aged Fischer 344 × Brown Norway (FBN) male rats. The FBN rat gastrocnemius muscle exhibits age-dependent atrophy, whereas the soleus muscle, up until 32 months, exhibits markedly fewer signs of atrophy. Both gastrocnemius and soleus muscles were analyzed, as well as plasma and urine. Compared to adult gastrocnemius, aged gastrocnemius showed evidence of reduced glycolytic metabolism, including accumulation of glycolytic, glycogenolytic, and pentose phosphate pathway intermediates. Pyruvate was elevated with age, yet levels of citrate and nicotinamide adenine dinucleotide were reduced, consistent with mitochondrial abnormalities. Indicative of muscle atrophy, 3-methylhistidine and free amino acids were elevated in aged gastrocnemius. The monounsaturated fatty acids oleate, cis-vaccenate, and palmitoleate also increased in aged gastrocnemius, suggesting altered lipid metabolism. Compared to gastrocnemius, aged soleus exhibited far fewer changes in carbohydrate metabolism, but did show reductions in several glycolytic intermediates, fumarate, malate, and flavin adenine dinucleotide. Plasma biochemicals showing the largest age-related increases included glycocholate, heme, 1,5-anhydroglucitol, 1-palmitoleoyl-glycerophosphocholine, palmitoleate, and creatine. These changes suggest reduced insulin sensitivity in aged FBN rats. Altogether, these data highlight skeletal muscle group-specific perturbations of glucose and lipid metabolism consistent with mitochondrial dysfunction in aged FBN rats.

**Electronic supplementary material:**

The online version of this article (doi:10.1007/s10522-014-9492-5) contains supplementary material, which is available to authorized users.

## Introduction

Sarcopenia describes the age-related decline in both lean body mass and skeletal muscle function. Skeletal muscle weakness in elderly subjects contributes to both reduced ability to perform usual activities of daily living and increased susceptibility to slip- and trip-induced falls (Janssen 2011; Landi et al. 2012). Resultant lack of independence and rehabilitation from falls represent significant aging-associated health economic burdens. As diagnostic criteria for sarcopenia continue to be evaluated, it will also be important to determine the molecular mechanisms that set sarcopenic muscle apart from otherwise healthy muscle in elderly subjects. Arriving at a precise causative model for sarcopenia has been complicated by its multifactorial etiology. Skeletal muscle function is dependent on neurological, neuromuscular, vascular, endocrine, and inflammatory systems which also decline with age. A second obstacle is predicting how environmental stimuli contribute to sarcopenia (e.g., acute muscle injury, surgery, reduced physical activity, nutritional status, mood). Perhaps most fundamental is our lack of understanding of specifically how myofibers adapt to the complex sequelae of aging.

Sarcopenia has historically been considered a disease of the terminally differentiated skeletal myofiber. In particular, glycolytic and oxidative ‘fast-twitch’ myosin heavy chain (MHC) type II-expressing myofibers preferentially atrophy and degenerate with age, compared to the exclusively oxidative, ‘slow-twitch’ MHC type I-expressing myofibers (Larsson et al. 1979; Joseph et al. 2012; Nilwik et al. 2013). Whole muscle groups are mixtures of fast and slow myofibers. In rats, the gastrocnemius muscle MHC protein pool comprises ~90 % MHC type II and 10 % MHC type I, while the soleus muscle comprises ~95 % MHC type I and 5 % MHC type II (Staron et al. 1999; Hepple et al. 2004; Russ et al. 2011). It is intriguing that co-expression of MHC type II may contribute significantly to slow myofiber and soleus atrophy in late old age (Snow et al. 2005; Purves-Smith et al. 2012). Muscle wasting may be further enhanced by a reduction in both the number and activity of regenerative satellite cells (Verdijk et al. 2012) and structural alterations of neuromuscular junctions (Li et al. 2011). The net physiological effect of aging on skeletal muscle is loss of strength and contractile function. Muscle contraction is dependent on the production of adenosine triphosphate (ATP) from one of three energetic reactions – anaerobic glycolysis, mitochondrial oxidative phosphorylation, and hydrolysis of creatine phosphate. Myosin ATPases use ATP to drive myosin-actin cross-bridge cycling and muscle contraction. With age, the type II myofiber exhibits diminished oxidative capacity and ATP content (Proctor et al. 1995; Drew et al. 2003). Age-related metabolic dysfunction is further evidenced by decreased mitochondrial autophagy and increased mitochondrial oxidative stress, leading to mitochondrial loss and myofiber apoptosis (Hagen et al. 2004; Chabi et al. 2008; Wohlgemuth et al. 2010). In both humans and rats, aged skeletal muscle tissue may adapt to type II myofiber loss through type I or mixed type I/II myofiber regeneration (Weber et al. 2012; Larsson et al. 1978). This shift toward type I myofiber repopulation generates an increasingly oxidative, and thus less energetically costly, metabolic profile in elderly subjects (Lanza et al. 2005; Hepple et al. 2004). These observations are consistent with a study showing that oxygen consumption and markers of mitochondrial oxidative capacity and biogenesis are increased in sedentary ‘high-functioning’ elderly subjects, yet decreased in sedentary ‘low-functioning’ elderly subjects (Joseph et al. 2012).

We sought to better understand muscle’s metabolic response to aging by comprehensive metabolomic profiling of gastrocnemius and soleus muscles from 15-month-old adult and 32-month-old aged Fischer 344 × Brown Norway (FBN) male rats. Age-related loss of skeletal muscle mass has been characterized in several rat inbred lines, and in particular the type II fiber-predominant gastrocnemius (Drew et al. 2003). The FBN rat strain in particular shows not only age-related changes in muscle weight and body composition comparable to humans, but also decreases in functional contractile strength (Rice et al. 2005; Hepple et al. 2004; Russ et al. 2013). We chose to compare muscles from 15-month-old adult and 32-month-old aged FBN rats to identify primary markers of sarcopenia. At 15 months, FBN rats show mild signs of muscle atrophy while severe sarcopenic pathology, including extensive fibrosis and muscle degeneration, appears beyond 32 months of age (Lushaj et al. 2008). We surveyed several skeletal muscle groups from FBN rats to guide choice of an atrophy-prone muscle, gastrocnemius, and a less atrophy-prone comparator, soleus, for metabolomic study. Described herein are metabolomic data from gastrocnemius, soleus, plasma, and urine of adult and aged FBN male rats. We also present a novel statistical workflow by which to differentially analyze ‘semi-quantitative’ versus ‘qualitative’ biochemicals within metabolomic datasets where data are ‘missing’ (i.e., a biochemical is not detected) preferentially from one experiment group. Altogether, these data highlight skeletal muscle group-specific metabolic dysfunction and adaptation in aged skeletal muscle.

## Methods

### Experimental animals

All experimental procedures were approved by the Institutional Animal Care & Use Committees at both The Ohio State University (Columbus, OH) and Abbott Laboratories (Chicago, IL). For the muscle morphometric survey, 6- and 32-month-old FBN F1 hybrid male rats were purchased from Harlan (Indianapolis, IN). Western blot and RT-PCR were performed on muscle from these mice, as previously described (Acharyya et al. 2005). Morphometric and molecular methods are also detailed in Online Resource 1. For metabolomic analyses, a separate cohort of FBN rats was used. Male 15- and 32-month-old FBN rats were purchased from Harlan. Upon arrival at The Ohio State University vivarium, rats were housed individually and provided water and food (Harlan 8640 Teklad Diet) ad libitum. Rats were housed for 12 days before being fasted for 16 h overnight prior to tissue collection. Blood and urine were collected with needle and syringe, and muscles were removed and trimmed of excess fat and connective tissue. Muscles were wrapped in aluminum foil, frozen in liquid nitrogen, and stored at −80 °C.

### Global metabolomics

Eight samples per aged and adult experimental groups were included in the metabolomic analysis of gastrocnemius, soleus, and plasma. Five and six urine specimens were analyzed from adult and aged rats, respectively. Metabolomic profiling was performed by Metabolon (Durham, NC, USA), and detailed methods are provided in Online Resource 1.

### Bioinformatics and statistics

#### Semi-quantitative analysis

Biochemicals had to meet a within-group 70 % fill threshold for semi-quantitative reporting of relative fold of change values and statistical analyses by methods otherwise routinely used for global ‘-omic’ profiling. Thus, if biochemicals were detected in at least 70 % of each group within a tissue of interest (e.g., at least 6 of the 8 soleus samples), the missing data were imputed with the lowest detectable value (1 × MIN) specific to the type of specimen, and a relative ratio was reported. For fold of change analysis, the values were scaled to the median intensity for that biochemical: (1) across the two age groups and across both muscle types, or (2) across the two age groups and within each of the plasma and urine datasets. The data were log-transformed for Welch’s two sample *t* test to compare data obtained from 15- and 32-month-old samples. Heat maps show fold of change values between 32-month-old aged and 15-month-old adult groups. Colored boxes represent statistically significant differences (*P* ≤ 0.05). Red signifies increased levels in aged muscle. Green signifies decreased levels in aged muscle. For plasma and urine candidate biomarker analysis, false discovery rate was estimated by *Q* values. Comparisons between age groups were taken as significant when *P* ≤ 0.05, and *Q* ≤ 0.10.

#### Qualitative analysis

For the minority of biochemicals that did not meet the semi-quantitative reporting threshold, a separate statistical analysis was applied as a requirement for qualitative reporting (e.g., reporting of detection percentage within age groups and display of boxplots). We considered 3 or more missing values from either group (representing 37.5 % or more of the available data of that group within muscle or plasma datasets) as subject to too much influence of an imputation assignment. In this case, a rule was applied: if all available values of one group were entirely above all available values of another group, and if there were at least one observation in one group and at least 6 observations in the second group, then this biochemical met the qualitative reporting requirement. The probability of ‘all of one group’ greater than ‘all of another group’ is <0.10 for all of the eligible combinations of non-missing numbers with these exceptions. If one group has only one non-missing value, or the groups are a combination of (4, 2) or (3, 2), the probability is >0.10. This analysis is shown in Online Resource 2. Note that this approach does not use the knowledge that a missing value is assumed to be too low to be measured and therefore lower than the values that are present.

## Results

### Muscle morphometry and markers of protein balance

To help determine choice of tissue for metabolomic studies, we carried out a morphometric survey of skeletal muscles from 6- and 32-month-old FBN rats (Online Resource 3). Between 6 and 32 months, average gastrocnemius wet weight decreased by 21.0 % (1.95 vs. 1.54 g), normalized weight decreased by 32.7 % (5.48 vs. 3.69 mg/g body weight), and myofiber cross-sectional area (CSA) decreased by 44.9 % (1,493 vs. 822 μM^2^). Soleus wet weight increased 12.1 % (141 vs. 158 mg) and myofiber CSA trended 12.4 % upward (780 vs. 877 μM^2^, *P* = 0.07). Plantaris showed no significant morphometric changes, and tibialis anterior showed a significant change only in normalized wet weight (1.91 vs. 1.55 mg/g body weight) between 6 and 32 months. It is noted, however, that differences between 6 and 32 months are not continuous, linear changes since body weight and some muscle weights increase through late middle age (Lushaj et al. 2008).

Western blot analysis of gastrocnemius lysates showed an age-related increase in the baseline, non-fasted levels of several markers of protein synthesis (i.e., activated insulin receptor, mTOR, and P70S6K1) (Online Resource 4). The highest molecular weight 4E-BP1 band also suggests increased protein synthesis with age through increased phosphorylation and inactivation of the translational repressor protein 4E-BP1 (Online Resource 4). No change in phospho-Akt1 expression was observed. Western blot also showed an increase in the activated form of NF-κB (phospho-p65), an upstream activator of proteasome-dependent protein degradation (Online Resource 4). RT-PCR showed increased expression of the protein degradation markers MuRF1 (muscle RING-finger protein-1 or TRIM63) and MAFbx (muscle atrophy F-box protein or atrogin-1 or FBXO32) (Bodine et al. 2001; Gomes et al. 2001) in aged gastrocnemius (Online Resource 4). These data confirm that the aged rat gastrocnemius serves as an accurate morphometric and molecular model of human sarcopenic muscle. Soleus was also chosen for metabolomic profiling to extend the analysis to an oxidative, type I fiber-predominant muscle group which appears less prone to atrophy in 32-month-old aged FBN rats.

### Global metabolic profile of skeletal muscle, plasma and urine

**Fig. 1:**
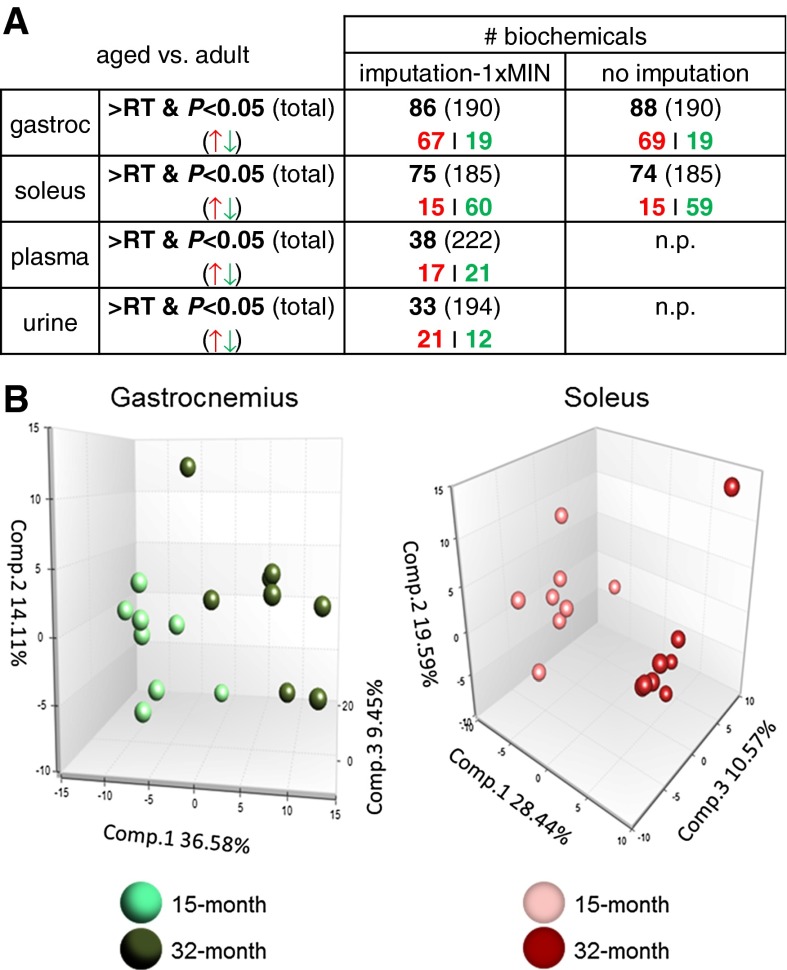
Overview of results from the metabolomic study. **a** Numbers of identified biochemicals are shown. *RT* 70 % filled value reporting threshold for semi-quantitative analysis (Methods), *P* *P* value determined by Welch’s *t* test of groups with or without imputation. **b** Principal components analysis of the muscle metabolomic matrices. *np* not performed

In total, we analyzed the global metabolic profile of gastrocnemius, soleus, and plasma from 15-month-old adult and 32-month-old aged rats (*n* = 8 per group). Five adult and six aged urine specimens were also analyzed. We detected 337 biochemicals in gastrocnemius, 327 in soleus, 357 in plasma and 461 in urine. Of these biochemicals, 220 biochemicals in gastrocnemius, 216 in soleus, 239 in plasma and 208 in urine matched known chemical structures in Metabolon’s chemical reference library. Of these known biochemicals, 190 biochemicals in gastrocnemius, 185 in soleus, 222 in plasma and 194 in urine met the reporting threshold for ‘semi-quantitative’ analysis (i.e., the biochemical was detected in at least 70 % of samples within each experimental group), and their fold of change values between 32- and 15-month-old samples are presented in Online Resources 5–7. Approximately 45 % of the biochemicals in gastrocnemius and soleus and 20 % of the biochemicals in plasma and urine showed significant age-related differences (Fig. 1a). The use of imputation methods had little effect on the muscle metabolomic signatures (Fig. 1a; Online Resource 8).

A separate min/max analysis for statistical significance was applied to ‘qualitative’ biochemicals for which >30 % of samples showed no detectable value within either age group (Methods; Online Resource 2). For example, despite only 3 detected values in one experimental group, xylulose does achieve statistical significance by this min/max method because all 8 observations in aged gastrocnemius were higher than the 3 observations in adult gastrocnemius (Online Resource 2). Altogether, the conservative semi-quantitative reporting threshold and min/max analysis for qualitative biochemicals were employed to limit imputation bias and focus interpretation on patterns from biochemicals within a detectable range. However, we cannot rule out that biochemicals below detection limits as well as those that do not meet the strict min/max test are relevant to aging, especially those that align within biological pathways implicated by semi-quantitative differences.

Principal component analysis (PCA) was used to provide a broad picture of how well aging explains differences in the muscle datasets. PCA analysis of the semi-quantitative metabolomic profiles separated age groups in both gastrocnemius and soleus (Fig. 1b). The first, second, and third components (i.e., 3 separate weighted sums of the original biochemicals) explain 36.6, 14.1, and 9.5 % of the variability of the biochemicals. Altogether, the first 3 components explain ~60.1 % of the variability of the original biochemicals. The list of most differentially abundant biochemicals with age (*P* < 0.05) shows very different profiles for gastrocnemius and soleus (Table 1). For example, 7 of the top 9 biochemicals more abundant at 32 months in gastrocnemius are related to carbohydrate metabolism. Comparing 15- and 32-month-old soleus, 2 TCA intermediates (i.e., fumarate and malate) are decreased with age along with several glycerophospholipids (GPLs) and acylcarnitines (Table 1; Online Resource 5). Of the 86 and 75 age-dependent biochemicals identified in gastrocnemius and soleus, respectively, 37 were shared and just 25 of these changed in the same direction (Online Resource 9), consistent with differential effects of aging on gastrocnemius and soleus.

**Table 1:**
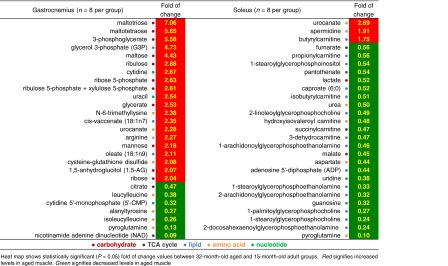
Metabolomic survey of most differentially abundant muscle biochemicals (32 vs. 15 months)

### Glucose metabolism in skeletal muscle

**Fig. 2:**
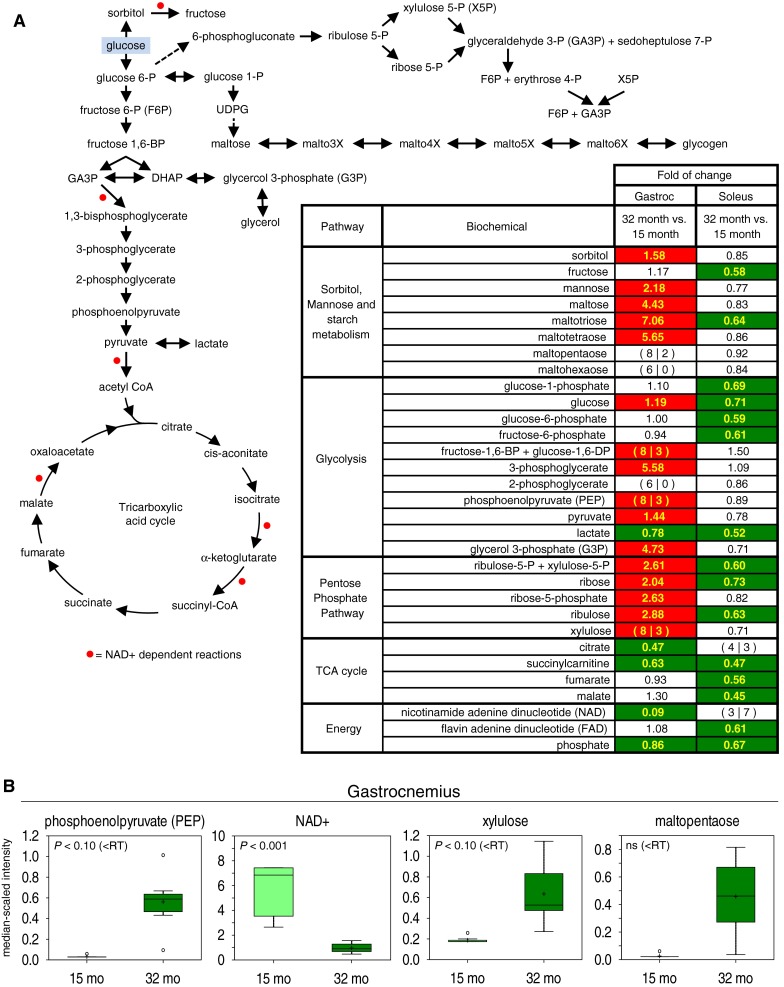
The fates of glucose are shown in the pathway through sorbitol, glycogen, the pentose phosphate pathway, glycolysis and the TCA cycle. **a** Heat map shows fold of change values between 32-month-old aged and 15-month-old adult groups within gastrocnemius and soleus datasets. *Colored boxes* represent statistically significant differences (*P* ≤ 0.05). *Red* signifies increased levels in aged muscle. *Green* signifies decreased levels in aged muscle. Where the semi-quantitative reporting threshold (RT) was not met for a biochemical (i.e., one group contained >2 samples with a missing value), the amount of samples showing a detectable value at 32-months (X) and 15-months (Y) are shown on the *left* and *right* sides within the parenthetical (X|Y). In this case, *colored background* denotes statistical significance in a min/max value test (Methods; Online Resource 2). **b** Boxplots are shown for key glucose metabolites and NAD in gastrocnemius. *Whiskers* represent max and min values, *vertical edges* *of* *box* represent upper and lower quartiles, + mean, *line* median, *unfilled circle* outlier, *ns* not significant, *RT* semi-quantitative reporting threshold

One of the most striking differences with age between muscle groups resided in levels of biochemical markers of glucose metabolism. The levels of several glycolytic intermediates increased to statistically significant levels in 32-month-old aged gastrocnemius (Fig. 2). For example, glucose is 19 % increased and 3-phosphoglycerate is greater than five-fold abundant in aged gastrocnemius (Fig. 2a). Though not meeting the semi-quantitative reporting threshold, both the (1) isobar of fructose-1,6-bisphosphate (FBP) and glucose-1,6-diphosphate and (2) phosphoenolpyruvate (PEP) are each detected in all 8 aged muscles but only detected in 3 adult muscles, both passing the min/max test for a statistically significant increase with aging (Fig. 2). Concomitant with the change in glycolytic intermediates during the aging process, there was a 91 % relative decrease in nicotinamide adenine dinucleotide (NAD) levels in aged gastrocnemius (Fig. 2). With respect to the TCA cycle, NAD depletion was also associated with a 44 % relative increase in pyruvate, yet a decrease in citrate (53 %) and succinylcarnitine (37 %), a surrogate marker of succinyl-CoA levels. Glucose fates alternative to glycolysis also showed changes with age. Sorbitol increased significantly with age (58 %) in gastrocnemius. Several pentose phosphate pathway intermediates (i.e., ribose, ribulose, and the sum of ribulose-5-phosphate and xylulose-5-phosphate) are also all increased greater than two-fold in aged gastrocnemius. While not meeting the semi-quantitative reporting threshold, xylulose did pass the min/max test for an increase with aging (Fig. 2b). The glycogen intermediates maltotetraose (5.65-fold), maltotriose (7.06-fold), and maltose (4.43-fold) also increased with age (Fig. 2a). Moreover, maltopentaose was detected in all 8 aged gastrocnemii but only 3 adult samples, however this comparison was not statistically significant (Fig. 2b). Several biochemicals show greater variability in the aged gastrocnemius, as represented by the boxplots for xylulose and maltopentaose (Fig. 2b).

On the other hand, the levels of several glycolytic intermediates [e.g., glucose, glucose-6-phosphate (G6P), and fructose-6-phosphate] decreased in aged versus adult soleus. Also opposite to gastrocnemius, soleus showed decreased levels of several pentose phosphate pathway intermediates (Fig. 2). Several TCA cycle intermediates were significantly reduced in aged soleus (e.g., fumarate, 44 %; malate, 55 %; succinylcarnitine, 53 %), and flavine adenine dinucleotide (FAD) was decreased 39 % compared to adult soleus.

### Fatty acid metabolism in skeletal muscle

**Fig. 3:**
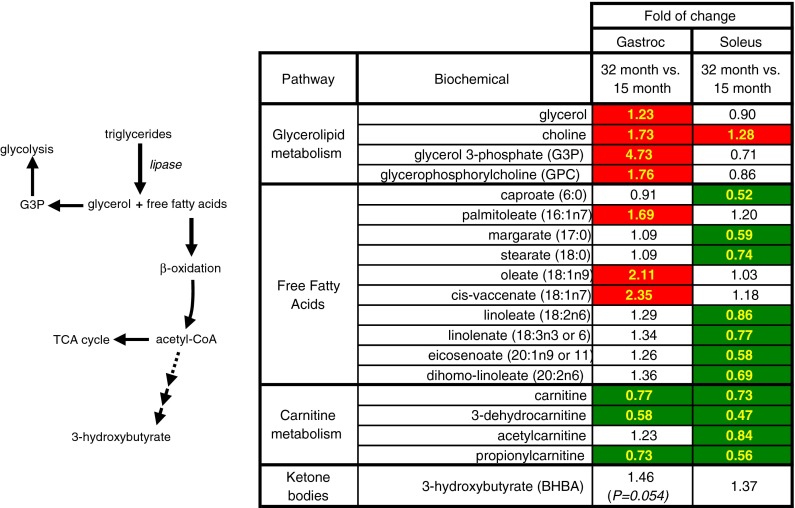
Lipolysis of fats results in the production of glycerol and free fatty acids. Heat map shows the statistically significant (*P* ≤ 0.05), positive (*red*) or negative (*green*), fold of change values between aged and adult muscle

Compared to adult gastrocnemius, aged gastrocnemius showed increased levels of glycerol (23 %), glycerol-3-phosphate (4.73-fold), and several monounsaturated free fatty acids (palmitoleate, 69 %; oleate, 2.11-fold; cis-vaccenate, 2.35-fold) (Fig. 3). 3-Hydroxybutyrate trended upward with age (46 %, *P* = 0.054). Aged gastrocnemius also showed increased choline (73 %) and glycerophosphocholine (76 %). Opposite to gastrocnemius, the soleus showed an age-related decrease in many fatty acids (e.g., margarate, 41 %; stearate, 26 %; linoleate, 14 %; linolenate, 23 %; and dihomo-linolenate, 31 %) (Fig. 3). Several GPLs were also decreased in aged soleus (Table 1; Online Resource 5). Both muscle groups showed reductions in carnitine (gastrocnemius, 23 %; soleus, 27 %) and low molecular weight acylcarnitines (long chain acylcarnitines were not measured) (Fig. 3).

### Amino acid metabolism in skeletal muscle

**Fig. 4:**
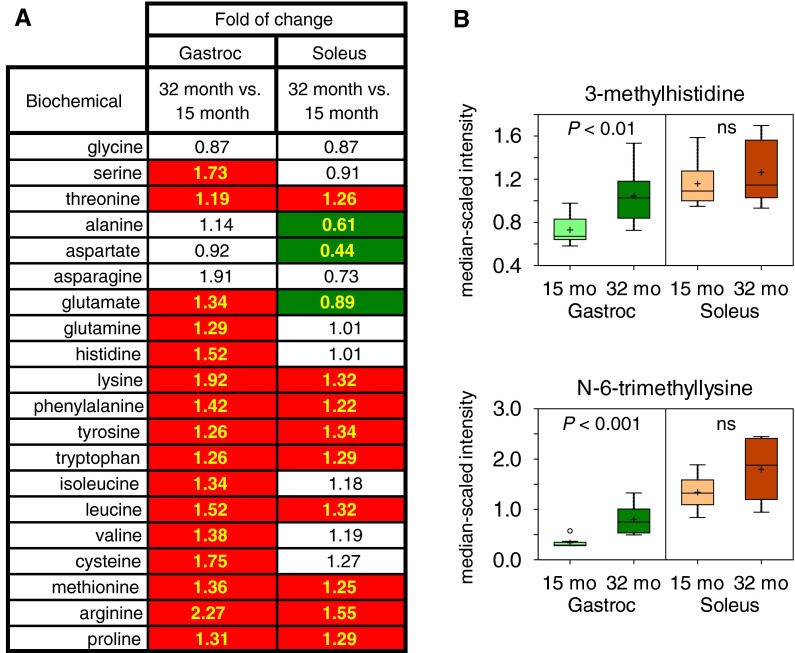
Increased levels of amino acids and indications of muscle degradation with age. **a** Heat map shows the statistically significant (*P* ≤ 0.05), positive (*red*) or negative (*green*), fold of change values between aged and adult muscle. **b** Boxplots are shown for the post-translationally modified amino acids, 3-methylhistidine and *N*_6_-trimethylysine. *Whiskers* represent max and min values, *vertical edges* *of* *box* represent upper and lower quartiles, + mean, *line* median, *unfilled circle* outlier, *ns* not significant

We detected 20 proteinogenic amino acids in muscle, and 16 increased significantly with age in gastrocnemius, and 9 increased in soleus with age (Fig. 4a). The post-translationally modified amino acids 3-methylhistidine and *N*_6_-trimethyl-lysine increased 43 % and 2.38-fold, respectively, in gastrocnemius with age (Fig. 4b). These amino acids have previously been characterized as markers of muscle damage (Lukaski et al. 1981). In addition, the dipeptides carnosine, carnitine, and anserine were all decreased with age in both muscle groups (Online Resource 5).

### Plasma and urine biochemicals associated with aging

A statistical method was employed to elucidate candidate plasma and urine biomarkers of aging. Biochemicals detected in plasma or urine with *P* value ≤0.05, *Q* value ≤0.10, and percent change >20 % between 15- and 32-month-old rats, were then ranked by fold of change (Table 2). These biochemicals serve as candidate biomarkers of aging in the FBN rat model. Of the 25 plasma biomarkers, 8 biochemicals also showed a significant change between aged and adult gastrocnemius, and with the exception of creatine, these changes were in the same direction. Of these 8 biochemicals, linear regression analysis of the aged samples alone showed that the levels of 3 biochemicals (i.e., 1,5-anhydroglucitol, 2′-deoxycytidine, and pyroglutamine) in plasma were significantly correlated with levels in gastrocnemius (*R* ^2^ > 0.70 and *P* < 0.05, Table 2). In particular, 1,5-anhydroglucitol (1-5-AG) showed the strongest correlation (*R* ^2^ = 0.77; *P* = 0.004) between plasma and gastrocnemius. Comparing aged and adult soleus, 1,5-AG levels were not significantly different. Of the 14 urine biomarkers, 4 biochemicals (i.e., C-glycosyltryptophan, hypotaurine, 3-methylhistidine, and 3-dehydrocarnitine) also showed a significant change with age in gastrocnemius. 3-methylhistidine (3-MeH) was increased 45 % in the urine from aged rats. As discussed prior, 3-MeH is a ‘dead-end’ metabolite that has been used clinically for decades as a urinary marker of various muscle catabolic conditions (Lukaski et al. 1981). 3-MeH levels are increased in aged gastrocnemius, but not different between aged and adult soleus (Table 2).

**Table 2:** Candidate plasma and urine biomarkers of aging

Plasma biomarker	Fold of change		
	Plasma	Gastroc	Soleus
Glycocholate	3.18	–	–
Heme	2.22	–	–
**1,5-Anhydroglucitol (1,5-AG)**	**2.19**	**2.07**	–
1-Palmitoleoylglycerophosphocholine	1.95	–	–
Palmitoleate (16:1n7)	1.64	1.69	–
Creatine	1.53	0.94	0.87
3-Methoxytyrosine	1.51	–	–
Alpha-hydroxyisovalerate	1.49	–	–
10-Heptadecenoate (17:1n7)	1.40	–	–
Threonine	1.35	1.19	1.26
Myristoleate (14:1n5)	1.28	–	–
Cis-vaccenate (18:1n7)	1.28	2.35	–
**2′-Deoxycytidine**	**1.22**	**1.76**	–
Urea	0.79	–	0.50
Creatinine	0.76	–	0.76
Deoxycarnitine	0.72	–	–
Pyridoxate	0.66	–	–
Proline-hydroxyproline	0.62	–	–
Hippurate	0.60	–	–
Threonate	0.53	–	–
3-Dehydrocarnitine	0.52	0.58	0.47
Aspartate	0.52	–	0.44
Pantothenate	0.52	–	0.54
3-Phenylpropionate	0.50	–	–
**Pyroglutamine**	**0.13**	**0.13**	0.10

Urine biomarker	Fold of change		
	Urine	Gastroc	Soleus
Imidazole lactate	3.44	–	–
3-Methyladipate	2.58	–	–
Azelate (nonanedioate)	2.00	–	–
Riboflavin (vitamin B2)	1.97	–	–
Hexanoylglycine	1.68	–	–
C-glycosyltryptophan	1.46	1.46	1.27
Pimelate (heptanedioate)	1.46	–	–
Hypotaurine	1.45	1.94	–
3-Methylhistidine (3-MeH)	1.42	1.43	–
Erythronate	1.41	–	–
*N*-acetylhistidine	0.70	–	–
3-Dehydrocarnitine	0.52	0.58	0.47
Suberate (octanedioate)	0.51	–	–
*N*-acetylleucine	0.45	–	–

Biochemicals shown met the requirements *P* < 0.05, *Q* < 0.10, and >20 % relative change between 32 and 15 months. – denotes biochemical was not significantly different between age groups, *boldface* indicates statistically significant correlation (*R* ^2^ > 0.70 and *P* < 0.05) of plasma biomarker with gastrocnemius levels within individual aged rats (*n* = 8)

### Integrative metabolomics–skeletal muscle aging in rat versus mouse models

**Fig. 5:**
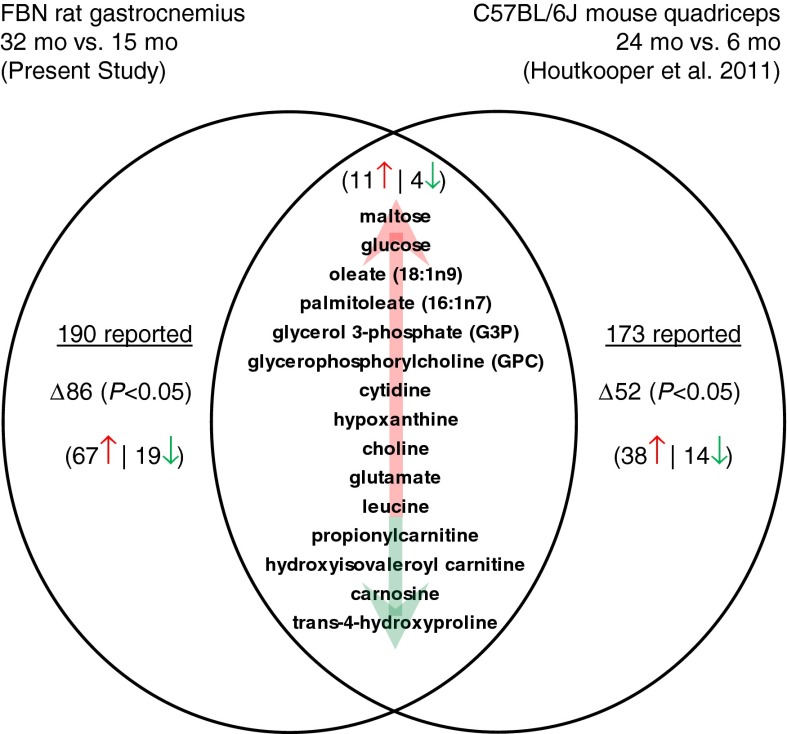
Venn diagram comparing the age-related FBN rat gastrocnemius metabolome reported in this study to the C57BL/6J quadriceps metabolome (Houtkooper et al. 2011)

In an effort to test for species translatability of these metabolomic data, we searched for published reports that also described metabolomic profiles of skeletal muscle aging. In particular, we searched for datasets that both investigated a sarcopenic muscle and also performed the analysis using Metabolon’s whole-metabolome profiling platform. To the best of our knowledge, the one published study that met these criteria described metabolomic differences in quadriceps muscles from 6- and 24-month-old C57BL/6J male mice (Houtkooper et al. 2011). Since biochemical intensity values were not reported, we applied the Welch’s *t* test to the reported fold of change values reported in the data supplement. In this mouse study, 173 known biochemicals were reported, with 52 showing significant differences. These data were compared to the metabolomic data described in this study of 15- and 32-month-old FBN rat gastrocnemius muscles. Across both studies, a total of 15 differentially abundant biochemicals representing several different metabolic pathways were reported in common (Fig. 5).

## Discussion

Sarcopenia describes the age-related loss of lean body mass and skeletal muscle strength. The multi-factorial etiology of sarcopenia has challenged attempts to both establish a clinical definition and understand causative molecular mechanisms. To better understand the effects of aging on skeletal muscle, we generated comprehensive metabolomic profiles from the gastrocnemius, soleus, plasma, and urine of 15-month-old adult and 32-month-old aged FBN male rats. Rat gastrocnemius muscle is a well-validated model of human sarcopenic muscle, showing age-related reductions in both mass and strength (Rice et al. 2005). Relative to adult gastrocnemius, aged gastrocnemius showed evidence of altered glucose metabolism, including accumulation of glycolytic, glycogenolytic, and pentose phosphate pathway intermediates. In contrast, aged soleus showed reductions in glycolytic intermediates, and overall, fewer age-related increases in biochemicals compared to gastrocnemius.

Aged gastrocnemius showed a metabolomic signature consistent with mitochondrial dysfunction. In particular, several tricarboxylic acid (TCA) cycle intermediates and cofactors were reduced with age. Citrate, succinylcarnitine (marker for succinyl-CoA), and NAD were all decreased in aged gastrocnemius, relative to adult (Fig. 2). NAD is a critical cofactor for TCA cycling and mitochondrial biogenesis. Decreased NAD levels and NAD/NADH redox ratio were also observed in skeletal muscles of aging mice and monkeys, respectively (Gomes et al. 2013; Pugh et al. 2013). NAD-dependent pyruvate decarboxylation to acetyl CoA immediately precedes the TCA cycle, so it is interesting that pyruvate was increased with age (Fig. 2). Acetyl CoA supply may thus be limited under conditions of NAD depletion (NADH and acetyl CoA were not measured). Citrate synthase activity may also be reduced, possibly as a negative feedback response to mitochondrial dysfunction. Several mitochondrial enzymes including citrate synthase were less abundant in aged FBN rat and human skeletal muscle (Hagen et al. 2004; Short et al. 2005). These data suggest that TCA cycling and ATP production are impaired in aged, atrophying muscle.

Since this study was carried out on overnight fasted rats, aged gastrocnemius may adapt acutely by shifting dysfunctional carbohydrate metabolism toward beta-oxidation of fatty acids. Aged gastrocnemius showed a metabolomic signature suggestive of increased lipolysis of fatty acids (Fig. 3). The increased levels of free fatty acids, however, suggest that beta-oxidation was impaired, possibly due to mitochondrial abnormalities. Metabolomic studies of mouse and human sera have also suggested incomplete fatty acid oxidation with age (Tomás-Loba et al. 2013; Yu et al. 2012). Alternatively, accumulation of free fatty acids may arise from increased adipose infiltration, de novo fatty acid biosynthesis, or lipid droplets (triglycerides were not measured). In humans, intramyocellular lipid droplets increase with age and are negatively associated with muscle strength (Conte et al. 2013). Increased lipid droplet size is also seen within skeletal myofibers of aged, yet pre-sarcopenic monkeys (Pugh et al. 2013). The overabundance of 16 of the 20 measured canonical proteinogenic amino acids in aged gastrocnemius suggests increased protein catabolism to free amino acids, possibly as a source of acetyl CoA for TCA cycling. It has already been shown that the ubiquitin proteasome pathway is more active in 30-month-old gastrocnemius relative to 4-month-old rats (Altun et al. 2010), and this pathway may be hyperactivated with age in the fasting state. Our molecular data from 6- to 32-month-old rat gastrocnemius confirm that protein degradation is increased with age, but also that protein synthesis is increased (Online Resource 4), perhaps to counterbalance protein degradation and presumably leading to a physiological state of dysregulated protein and energy balance. These data also fit with and may be exacerbated by the larger picture of desensitized insulin signaling through which the activated insulin receptor continuously cues muscle protein synthesis by inactivating the translation repressor 4E-BP1 (Online Resource 4).

Glycolysis is severely perturbed in aged gastrocnemius, which shows an overabundance of several downstream glycolytic intermediates (Fig. 2). These data suggest impaired glycolysis since glycolytic enzymes are typically efficient and rapidly convert excess glucose to pyruvate (Milne et al. 2002). In particular, the chromatographic isobar of glucose-1,6-diphosphate and FBP is detected in just 3 adult gastrocnemius samples, but all of the aged samples. This could be partly explained by decreased activity of glyceraldehyde-3-phosphate dehydrogenase (GAPDH), which catalyzes the NAD-dependent formation of 1,3-bisphosphoglycerate from glyceraldehyde-3-phosphate (GA3P), the direct downstream metabolite of FBP. GAPDH expression and activity have already been shown to be decreased in aged rat gastrocnemius (Lowe et al. 2000). Reduction of NAD may contribute to decreased GAPDH activity, GA3P accumulation and feedback inhibition of FBP catalysis by aldolase. PEP was also detected in more aged gastrocnemius samples (Fig. 2). Pyruvate kinase transfers the phosphate from PEP to ADP to form ATP and pyruvate. This reaction is positively regulated by both excess upstream FBP and its own substrate PEP (Fenton and Hutchinson 2009). In this study, however, these positive effectors appeared unable to promote pyruvate kinase activity in aged rat gastrocnemius. It is interesting that several free amino acids, including phenylalanine which was more abundant in aged gastrocnemius (Fig. 4), are allosteric inhibitors of pyruvate kinase (Williams et al. 2006). In such a way, free amino acids could promote beta-oxidation during catabolic states accompanied by glycolytic dysfunction. Pyruvate also accumulated in aged gastrocnemius (Fig. 2), suggesting that pyruvate dehydrogenase expression or activity is compromised. It is interesting that the age-related decreases in the expression of pyruvate dehydrogenase and 6-phosphofructo-2-kinase/FBP phosphatase are correlated with decreased muscle mass in Sprague–Dawley (SD) rats (Ibebunjo et al. 2013). It is not clear whether downregulation of glycolytic enzymes precedes, contributes to, or follows from mitochondrial dysfunction in aged muscle.

Intracellular glucose has alternate metabolic fates which appear to be differentially utilized in aged rat gastrocnemius. Decreased glycolysis permits both the packaging of glucose into glycogen and conversion to sorbitol. In this study, glucose metabolism shifted toward sorbitol and pentose pathways (Fig. 2). There was also evidence of severe perturbation of glycogen metabolism with age. Rather than isomerization to fructose, G6P can be converted to glucose-1-phosphate (G1P) for concatamerization and branching by a suite of enzymes to create glycogen. Glycogen was not directly measured, but we do report overabundance of several malto-oligosaccharides—intermediates of glycogen metabolism—in aged rat gastrocnemius relative to adult. The question remains whether elevated malto-oligosaccharides denote increased glycogen synthesis or degradation. Age-related increases in glucose, maltose, and malto-oligosaccharides (Fig. 2) were similarly observed in a metabolomic study of quadriceps muscles from 6- and 24-month-old C57BL/6J mice (Houtkooper et al. 2011) (Fig. 5). Perhaps initial de-branching steps of glycogenolysis are activated in aged, fasted muscle, but phosphorylase activity is inhibited and unable to catabolize free oligosaccharides. An age-related decrease in glycogen phosphorylase activity has been described in the tibialis muscle of F344 rats (Montori-Grau et al. 2009), and UDP-glucose phosphorylase-2 expression is positively correlated with muscle mass in SD rats (Ibebunjo et al. 2013). Defects in the aging lysosomal compartment may also contribute to incomplete digestion of glycogen. Consistent with this interpretation, glycogen content decreases with age in a study of rectus abdominalis muscles from FBN rats (Gupta et al. 2000). Other reports do not agree on effects of aging on muscle glycogen content (Montori-Grau et al. 2009). Nonetheless, our data strongly suggest that glucose and glycogen handling are significantly altered in sarcopenic muscle.

NAD depletion in aged gastrocnemius may result from and further contribute to the glycolytic and mitochondrial defects previously discussed. NAD is a critical electron transporter and enzyme cofactor throughout mitochondrial respiration and oxidative phosphorylation. NAD also serves as a substrate for both the sirtuin family of NAD-dependent histone deacetylases and the DNA repair enzyme, poly(ADP-ribose) polymerase (PARP). Aging-related, progressive increase in cellular oxidative damage drives NAD-consuming PARP activity, thus limiting NAD supply for respiration and longevity-promoting sirtuin activities (Pillai et al. 2005; Massudi et al. 2012). PARP may indeed be a large cellular consumer of NAD since skeletal muscle of PARP1-deficient mice display increased NAD content, sirtuin expression, and mitochondrial biogenesis (Bai et al. 2011). In another study of liver, heart, kidney, and lung tissues from 3-, 12-, and 24-month-old Wistar rats, oxidative damage and PARP activity progressively increase with age, while antioxidant capacity, NAD content, and sirtuin-1 activity progressively decline (Braidy e et al. Braidy et al. 2011). Restoring NAD is thus a candidate approach for mitigating age-related functional decline in skeletal muscle. Dietary augmentation of NAD with nicotinamide mononucleotide increased ATP levels and promoted an oxidative profile in gastrocnemius of aged mice (Gomes et al. 2013). Moreover, nicotinamide riboside supplementation in mice fed a high-fat diet increased NAD content in several tissues including skeletal muscle, with evidence for increased mitochondrial abundance, activation of sirtuin target genes, and improved treadmill running endurance (Canto et al. 2012).

Whereas gastrocnemius begins to atrophy just beyond middle age in FBN rats, the soleus does not atrophy until much older age (Purves-Smith et al. 2012), and may partly explain why soleus shows a very different metabolomic signature of aging between 15 and 32 months (Table 1; Online Resource 5). Nonetheless, sub-optimal bioenergetics was indicated. Fumarate and malate are decreased in aged soleus relative to adult soleus, and the decrease in the cofactor FAD is predicted to compromise succinate dehydrogenase activity. Aged soleus showed decreased levels of glycolytic and pentose phosphate pathways intermediates, suggesting that glycolysis and salvage pathways are adaptively upregulated to generate energy in the fasted state. In parallel, free fatty acids and acylcarnitines are also decreased in aged soleus. These data suggest that aged soleus myofibers do contain functional mitochondria, and that the supply or storage of carbohydrates and lipid substrates may be the limiting energetic factor.

Several age-related metabolomic changes in muscle are specific to the soleus. For example, several GPLs decrease in aged soleus (Online Resource 5). GPLs are saturated long-chain glycerophospho-ethanolamines and -cholines created by the phospholipase-mediated hydrolysis of polyunsaturated phospholipids. GPLs are critical structural components of the plasma membrane and promote positive, or fluidizing, curvature to membranes (Fuller and Rand 2001). These data suggest that soleus may adapt to aging by shifting its plasma and organellar membrane lipidomic profiles over time, or in the fasted state, by acutely catabolizing GPLs for energy. It is intriguing that many GPLs are also lowered in elderly human sera samples compared to younger subjects (Collino et al. 2013). Blood plasma GPLs and other lipids are also associated with ‘familial longevity’ in middle-aged offspring of human nonagenarians (Gonzalez-Covarrubias et al. 2013). However, it is not yet known how the plasma lipidome correlates with and influences the muscle lipidome, contractile function, and aging.

Analysis of candidate plasma and urine biomarkers of aging suggest a physiological state of altered glucose handling and insulin resistance in aged rats. The age-related increase of plasma monounsaturated fatty acids, such as palmitoleate (16:1n7) and cis-vaccenate (18:1n7), suggest increased lipolysis of triglycerides and reduced uptake by tissues, consistent with insulin resistance (Table 2; Online Resource 6). 1,5-Anhydroglucitol (1,5-AG) is also elevated with aging in plasma and gastrocnemius (Table 2). Serum 1,5-AG is inversely correlated with diabetes and 2-h postprandial glucose levels in humans (Yamanouchi et al. 1987; Stettler et al. 2008). 1,5-AG is the naturally occurring, metabolically stable 1-deoxy form of glucose which, during hyperglycemia, is outcompeted by glucose for renal tubular reabsorption (Kim and Park 2013). Elevated plasma 1,5-AG in aged, fasted rats may reflect insulin resistance or dysfunctional renal handling of glucose. The plasma marker creatine is also increased in aged rats, likely due to skeletal muscle ‘leak,’ as creatine is decreased in both aged muscle groups (Table 2). Reduced plasma tryptophan in aged rats (Online Resource 6) reflects similar observations in metabolomic studies of human sera samples (Yu et al. 2012; Collino et al. 2013). Reduced plasma and muscle levels of free proline-hydroxyproline (Table 2), a dipeptide constituent of collagen, may reflect less turnover and increased deposition and cross-linking of collagen during aging. Gastrocnemius trans-4-hydroxyproline, another amino acid modification common to collagen, is also reduced with age (Table 2). Increased collagen deposition and cross-linking promotes muscle stiffness and reduces joint mobility in elderly subjects, and joint mobility is even more limited in elderly subjects with diabetes (Abate et al. 2011). In urine and muscle, the age-associated increase in C-glycosyltryptophan (Table 2) may indirectly reflect increased glycosyltransferase activity against mature proteins. Alternatively, C-glycosyltryptophan may be an enzyme-independent advanced glycation product similar to carboxymethyllysine (CML). CML was associated with slow walking speed in a study of elderly subjects (Semba et al. 2010) and decreased grip strength in community-dwelling elderly women (Dalal et al. 2009).

It is important to note several caveats regarding interpretation of metabolomic data within this study. The FBN rats used herein were fasted for 16 h overnight prior to collection of tissue. Fasting is the standard method to physiologically normalize basal metabolism across experimental groups, but overnight fasting is itself a strong experimental stimulus. In young SD rats, 16 h overnight fasting caused changes in up to 50 % of the metabolome in serum, urine, and liver, compared to age-matched rats given ad libitum access to food (Robertson et al. 2011). Decreased serum glucose in fasted rats was also associated with significant changes in the expression of liver genes related to glucose metabolism. Thus, the metabolomic differences that we report herein may not necessarily be observed postprandially or throughout ad libitum feeding. A second critical consideration is that skeletal muscle is a heterogeneous tissue. Not only are muscle groups comprised of many cell types, but mature myofibers themselves exist in several metabolic varieties with varying enzymatic rates. The rat gastrocnemius is predominantly comprised of type II myofibers, whereas the soleus predominantly contains type I myofibers (Staron et al. 1999). In type II fiber-predominant muscle groups like the gastrocnemius, ATP is largely derived from glycogen and glucose, which are anaerobically processed to pyruvate and lactate in the cytosol or oxidatively processed through mitochondria. Type I fiber-predominant muscles like the soleus contain more mitochondria for almost exclusive oxidative metabolism. Type I myofibers are thus better primed for TCA cycling of citrate through initial reaction with acetyl CoA derived either from pyruvate or catabolism of fatty acids and proteins. Thus, metabolic changes in whole muscle groups—especially aged muscle—reflect a complex a mixture of cells. While 32-month-old muscles were not tested for MHC content or fiber type distribution in this study, 28- to 29-month-old gastrocnemius and 30-month-old soleus samples showed little change in MHC content compared to younger samples of the same muscles (Hepple et al. 2004; Snow et al. 2005). A final caveat is that we do not know the effect of acute differential cage activity the night before tissue harvest on muscle metabolomic profiles.

These metabolomic data show that aged, fasted rats exhibit signs of insulin resistance and compromised muscle energetics. Reduced NAD content in aged muscle suggests that ATP is also reduced. While ATP was not measured in this study, isolated mitochondria from the gastrocnemius of 26-month-old F344 rats produce 50 % less ATP than 12-month-old controls (Drew et al. 2003). In addition, a high-resolution in situ respirometry study of mouse gastrocnemius, and not soleus, showed decreased mitochondrial efficiency with aging (Jacobs et al. 2013). Similar data were generated in aged FBN rats, however mitochondrial function did not explain the extent of atrophy between muscle groups (Picard et al. 2011). Beyond mitochondrial function, type II myofibers may be compromised by their energetic reliance on glucose, whose metabolism, indirectly based on our metabolomic data from gastrocnemius, is clearly dysregulated with age. It would be interesting to test whether differential processing of intramyocellular lipid droplets contributes to myofiber type specific pathologies. Aging-associated changes specific to type II myofiber atrophy may also include a differential response to decreased physical activity or neuromuscular changes that impact sarcoplasmic reticulum function and excitation–contraction coupling. Slow type I myofibers may be less affected by age-related denervation and insulin resistance. It is intriguing that type I myofiber clustering, a marker of regeneration, occurs differentially in aged rats and is associated with greater muscle strength (Weber et al. 2012). In human elderly subjects, markers of mitochondrial activity and oxidative capacity are associated with ‘high functioning’ status (Joseph et al. 2012). Further experimentation is needed to test causation between altered muscle metabolism and age-related muscle atrophy.

## Electronic supplementary material

Below is the link to the electronic supplementary material.

Supplementary material 1 (PDF 72 kb)

Supplementary material 2 (PDF 32 kb)

Supplementary material 3 (PDF 37 kb)

Supplementary material 4 (PDF 346 kb)

Supplementary material 5 (XLSX 54 kb)

Supplementary material 6 (XLSX 43 kb)

Supplementary material 7 (XLSX 44 kb)

Supplementary material 8 (PDF 38 kb)

Supplementary material 9 (PDF 100 kb)
