# Supplementary material for: Metabolomic profiling reveals severe skeletal muscle group-specific perturbations of metabolism in aged FBN rats
Source: Biogerontology. 2014 Mar 21;15(3):217–32. doi: 10.1007/s10522-014-9492-5 (PMC4019835; doi:10.1007/s10522-014-9492-5)
Supplement: Supplementary file 1 — Supplementary material 1 (PDF 72 kb) [file 10522_2014_9492_MOESM1_ESM.pdf]

S.M. Garvey[1] · J.E. Dugle[1] · A.D. Kennedy[2] · J.E. McDunn[2] · W. Kline[3] · L. Guo[2] · D.C. Guttridge[3] · S.L. Pereira[1] · N.K. Edens[1]
[1]Abbott Nutrition R&D, Columbus, OH · [2]Metabolon, Inc., Durham, NC · [3]The Ohio State University, Columbus, OH

Online Resource 1
Experimental Procedures

Experimental animals for muscle morphometric survey

All experimental procedures were approved by the Institutional Animal Care & Use Committees at both The Ohio State University (Columbus, OH) and Abbott Laboratories (Chicago, IL). For the skeletal muscle morphometric survey, 6-month and 32-month-old Fischer 344 X Brown Norway (FBN) F1 hybrid male rats were purchased from Harlan (Indianapolis, IN). Upon arrival at the The Ohio State University Biomedical Research Tower (Columbus, OH), rats were housed individually and provided water and food (Harlan 8640 Teklad Diet) ad libitum for 7 days before necropsy. Rats were weighed and then euthanized by carbon dioxide asphyxiation. Gastrocnemius, plantaris, soleus, and tibialis anterior muscles were removed and trimmed of any excess fat or connective tissue. For histological analysis, muscles were mounted onto parafilm-sealed cork board and then covered with optimal cutting temperature (OCT) compound. The specimen was then immersed in liquid nitrogen cooled 2-methylbutane for up to 45 seconds. OCT compound-embedded samples were covered in foil and stored at -80 °C until day of sectioning.

Myofiber CSA analysis

OCT compound-embedded samples were cut at 10-μM on a Leica Microsystems (Wetzlar, Germany) CM1950 cryostat. All sections for histological analysis were derived from the midbelly of muscles. A portion of sections were stained with hematoxylin and eosin, cleared with xylene and cover-slipped using standard protocols. For myofiber cross-sectional area analysis, slides were imaged at 10X magnification with an Olympus (Center Valley, PA) BX51 microscope and analyzed with Olympus MicroSuite™ FIVE imaging software.

Western blot and RT-PCR

Western blots and RT-PCR were performed as previously described (Acharyya et al. 2005) with the following antibodies: anti-phospho-p65 (sc-3033, Santa Cruz Biotechnology, Inc., Dallas, Texas), anti-phospho-IR (407707, Calbiochem/Merck KGaA, Darmstadt, Germany), anti-phospho-Akt (9271, Cell Signaling Technologies, Inc., Danvers, Massachussetts), anti-Akt (9272, Cell Signaling), anti-phospho-mTOR (2976, Cell Signaling), anti-mTOR (2972, Cell Signaling), anti-phospho-p70S6K (9206, Cell Signaling), anti-P70S6K (9202, Cell Signaling), and anti-4E-BP1 (9452, Cell Signaling). RT-PCR primers included:
MuRF1 forward: 5' - GGA CGG AAA TGC TAT GGA GAA CC - 3'
MuRF1 reverse: 5' - GAT GGC TGT TTC CAC AAG CTT GG - 3'
MAFbx forward: 5' - CCT CAG CAG TTA CTG CAA CAA GG - 3'
MAFbx reverse: 5' - CAT CTT CTT CCA ATC CAG CTG CC - 3'
GAPDH forward: 5' - CAC GGC AAA TTC AAC GGC ACA GTC AAG G - 3'
GAPDH reverse: 5' - GTT CAC ACC CAT CAC AAA CAT GG - 3'

S.M. Garvey[1] · J.E. Dugle[1] · A.D. Kennedy[2] · J.E. McDunn[2] · W. Kline[3] · L. Guo[2] · D.C. Guttridge[3] · S.L. Pereira[1] · N.K. Edens[1]
[1]Abbott Nutrition R&D, Columbus, OH · [2]Metabolon, Inc., Durham, NC · [3]The Ohio State University, Columbus, OH

Global metabolomics

Metabolomic profiling of gastrocnemius, soleus, plasma, and urine from adult and aged rats was performed by Metabolon (Durham, NC, USA) according to published methods (Evans et al. 2009). Upon receipt at Metabolon, samples were extracted and normalized by weight. For example, 100 mg of tissue was extracted with 450 µL of extraction solvent. Therefore, one can compare each sample of solid tissue because the mass was normalized by how much extraction solvent was utilized. After the extraction of biochemicals from gastrocnemius, soleus, plasma, and urine, the extracts were analyzed by GC/MS and LC/MS. We performed chromatographic separation, followed by full-scan mass spectroscopy, to record and quantify all detectable ions presented in the samples. We identified biochemicals with known chemical structure by matching the ions' chromatographic retention index and mass spectral fragmentation signatures with reference library entries created from authentic standard biochemicals under the identical analytical procedure as the experimental samples (Evans et al. 2012). Raw area counts for each biochemical in each sample were normalized to correct for variation resulting from instrument interday tuning differences. Raw area counts for a compound were divided by the median value, setting the medians equal for each day's run. Missing values were assumed to result from areas being below the limits of detection. Where not stated otherwise, missing values for a given biochemical were imputed with the observed minimum after the normalization step. Semi-quantitative values were derived from integrated raw detector counts of the mass spectrometers. Importantly, while peak area comparisons between samples represent relative amounts of each ion detected, different compounds and ions have different ionization potentials. To preserve all of the variation, yet allow compounds of widely different raw peak areas to be compared directly on a similar graphical scale, the normalized intensities were scaled by their median values for each compound. In sum, 8 samples per experimental group were included in the analysis of gastrocnemius muscle, soleus muscle, and plasma, while 5 and 6 urine specimens were analyzed from adult and aged rats, respectively.

Imputation test.

In the semi-quantitative analysis of biochemicals meeting the reporting threshold, imputation at 1xMIN assumes the likelihood that more values were 'near misses' to the quantification limits than were actual zeros. However, imputation at 1xMIN can distort P-values. For this reason, imputation at 0.5xMIN and MIN/SQRT(2) was tested, assuming that the portion of non-measurable values is approximated by a lower tail of the entire distribution of values. Imputation at 0.5xMIN and MIN/SQRT(2) approximated the probability profile attained with standard imputation at 1xMIN (data not shown). In a second test of the effects of imputation, imputation was outright not used and missing values were not included in the analysis (Online Resource 7). Because the probability profile without imputation approximated that with imputation, we proceeded to report relative ratios and probabilities with the standard 1xMIN imputation method.

S.M. Garvey[1] · J.E. Dugle[1] · A.D. Kennedy[2] · J.E. McDunn[2] · W. Kline[3] · L. Guo[2] · D.C. Guttridge[3] · S.L. Pereira[1] · N.K. Edens[1]
[1]Abbott Nutrition R&D, Columbus, OH · [2]Metabolon, Inc., Durham, NC · [3]The Ohio State University, Columbus, OH

References

Acharyya S, Butchbach ME, Sahenk Z, Wang H, Saji M, Carathers M, Ringel MD, Skipworth RJ, Fearon KC, Hollingsworth MA, Muscarella P, Burghes AH, Rafael-Fortney JA, Guttridge DC (2005) Dystrophin glycoprotein complex dysfunction: a regulatory link between muscular dystrophy and cancer cachexia. Cancer Cell 8:421-432

Evans AM, DeHaven CD, Barrett T, Mitchell M, Milgram E (2009) Integrated, nontargeted ultrahigh performance liquid chromatography/electrospray ionization tandem mass spectrometry platform for the identification and relative quantification of the small-molecule complement of biological systems. Anal Chem 81:6656-6667

Evans AM, Mitchell WM, Dai H, DeHaven CD (2012) Categorizing ion –features in liquid chromatography/mass spectrometry metobolomics data. Metabolomics 2:110
