# Supplementary material for: Metabolomic profiling reveals severe skeletal muscle group-specific perturbations of metabolism in aged FBN rats
Source: Biogerontology. 2014 Mar 21;15(3):217–32. doi: 10.1007/s10522-014-9492-5 (PMC4019835; doi:10.1007/s10522-014-9492-5)
Supplement: Supplementary file 2 — Supplementary material 2 (PDF 32 kb) [file 10522_2014_9492_MOESM2_ESM.pdf]

Metabolomic profiling reveals severe skeletal muscle group-specific perturbations of metabolism in aged FBN rats

S.M. Garvey[1] · J.E. Dugle[1] · A.D. Kennedy[2] · J.E. McDunn[2] · W. Kline[3] · L. Guo[2] · D.C. Guttridge[3] · S.L. Pereira[1] · N.K. Edens[1]
[1]Abbott Nutrition R&D, Columbus, OH · [2]Metabolon, Inc., Durham, NC · [3]The Ohio State University, Columbus, OH

Online Resource 2 Min/max analysis of 'qualitative' metabolites not meeting 'semi-quantitative' reporting threshold

Question (Q): In comparisons where there are at least one observation in one group and at least 6 observations in the second group, are all values of the 32-month aged group higher than all values of the 15-month adult group (and vice versa)? This question is answered in the final column, and if yes, the biochemical achieves statistical significance (P <0.1). n, number of sample detected within a group for a given assay; min, minimum raw value within a group; max, maximum raw value within a group; 15, 15-month adult group; 32, 32-month aged group, ***bold face and yellow indicates that the biochemical passes min/max test for statistical significance***

Muscle Group	Biochemical	n15	n32	min32	max32	min15	max15	Q: ALL HIGH or ALL LOW?
gastroc	1-arachidonoylglycerophosphocholine	4	5	79130	683013	153921	406144	no
gastroc	1-linoleoylglycerophosphocholine	5	5	107734	778043	198381	644415	no
gastroc	2-linoleoylglycerophosphocholine	7	5	438784	1544544	99847	1681454	no
gastroc	3-indoxyl sulfate	4	7	4047	11222	4260	6624	no
gastroc	4-hydroxybutyrate (GHB)	5	1	9722	9722	8627	21087	no
gastroc	5-dodecenoate (12:1n7)	4	7	9641	37244	8408	29972	no
gastroc	7-beta-hydroxycholesterol	4	6	17370	41357	20929	63169	no
gastroc	adenosine 5'-diphosphate (ADP)	4	7	32753	498666	28712	105141	no
gastroc	allantoin	8	5	33369	72773	12253	62216	no
gastroc	beta-muricholate	4	5	6525	25455	4840	21236	no
gastroc	beta-sitosterol	5	7	42076	101000	46682	115611	no
gastroc	gamma-glutamylleucine	4	6	59702	84795	30261	83763	no
gastroc	heme	3	4	24685	95729	39365	57415	no
gastroc	inosine 5'-monophosphate (IMP)	8	5	32748	6724195	4412730	13574828	no
**gastroc**	**Isobar: fructose 1,6-diphosphate, glucose 1,6-diphosphate**	**3**	**8**	**18453**	**241774**	**5290**	**16484**	**yes**
gastroc	maltopentaose	2	8	33863	754110	19751	56603	no
**gastroc**	**phosphoenolpyruvate (PEP)**	**3**	**8**	**40590**	**437036**	**11939**	**25165**	**yes**
gastroc	phosphopantetheine	3	1	82379	82379	32950	70057	no
gastroc	pipecolate	3	5	33687	63842	45215	61667	no
gastroc	S-adenosylhomocysteine (SAH)	8	5	29753	58892	46517	109973	no
gastroc	sedoheptulose-7-phosphate	6	4	14260	43376	14959	63487	no
gastroc	squalene	7	4	61186	134609	104736	296470	no
**gastroc**	**xylulose**	**3**	**8**	**14422**	**60989**	**9295**	**13764**	**yes**
**soleus**	**1-arachidonoylglycerophosphocholine**	**6**	**1**	**98253**	**98253**	**99576**	**351564**	**yes**
soleus	1-linoleoylglycerophosphocholine	8	5	55170	330901	61627	1103166	no
soleus	1-oleoylglycerophosphocholine	8	4	70763	349075	27427	1099172	no
soleus	1,6-anhydroglucose	4	5	160305	334592	238189	430261	no
soleus	2-arachidonoylglycerophosphocholine	8	5	50751	480902	87664	1353253	no
soleus	2-docosapentaenoylglycerophosphoethanolamine	7	5	45996	1164976	198655	2723901	no
soleus	2-oleoylglycerophosphocholine	5	3	42706	186053	87630	283794	no
soleus	2-oleoylglycerophosphoethanolamine	5	7	13062	31680	12757	56814	no
soleus	2-palmitoylglycerol (2-monopalmitin)	5	1	108679	108679	110237	165799	no
soleus	2-palmitoylglycerophosphocholine	7	3	98312	398903	120903	536217	no
soleus	2-palmitoylglycerophosphoethanolamine	7	3	63867	548236	89836	445867	no
soleus	7-beta-hydroxycholesterol	5	3	8467	34887	17972	40301	no
soleus	alanylleucine	2	3	31426	43834	31358	57232	no
soleus	alanyltyrosine	2	7	38294	188597	71401	124753	no
soleus	allantoin	7	5	15683	33911	8814	49768	no
soleus	alpha-tocopherol	1	7	13928	184327	35046	35046	no
soleus	beta-sitosterol	7	5	57982	111794	52155	168006	no
soleus	citrate	3	4	5442	12342	5193	8773	no
**soleus**	**galactitol (dulcitol)**	**8**	**4**	**33107**	**58282**	**62830**	**121927**	**yes**
soleus	heme	6	5	39907	459899	24263	134986	no
soleus	N-acetylalanine	4	7	57121	97989	56850	85047	no
soleus	nicotinamide adenine dinucleotide (NAD+)	7	3	17280	31744	16690	52957	no
soleus	palmitoylcarnitine	8	4	38029	336689	75099	401826	no
soleus	pipecolate	5	6	31284	75313	49449	71648	no
soleus	pseudouridine	5	6	42903	98910	54119	83278	no
soleus	S-adenosylhomocysteine (SAH)	7	4	35569	59689	35973	94014	no
soleus	S-lactoylglutathione	7	5	17264	41076	9454	31833	no
soleus	sphingosine	7	3	49021	204028	102546	446495	no
soleus	squalene	8	2	45350	59861	53679	110864	no
