# Supplementary material for: Metabolomic profiling reveals severe skeletal muscle group-specific perturbations of metabolism in aged FBN rats
Source: Biogerontology. 2014 Mar 21;15(3):217–32. doi: 10.1007/s10522-014-9492-5 (PMC4019835; doi:10.1007/s10522-014-9492-5)
Supplement: Supplementary file 3 — Supplementary material 3 (PDF 37 kb) [file 10522_2014_9492_MOESM3_ESM.pdf]

Metabolomic profiling reveals severe skeletal muscle group-specific perturbations of metabolism in aged FBN rats
S.M. Garvey[1] · J.E. Dugle[1] · A.D. Kennedy[2] · J.E. McDunn[2] · W. Kline[3] · L. Guo[2] · D.C. Guttridge[3] · S.L. Pereira[1] · N.K. Edens[1]
[1]Abbott Nutrition R&D, Columbus, OH · [2]Metabolon, Inc., Durham, NC · [3]The Ohio State University, Columbus, OH

Online Resource 3 Characterization of male FBN rats used in the morphometric survey and comprehensive metabolomic studies

Morphometric Survey	6 months (n=5)	32 months (n=5)	% change	*P*-value
fed body weight (g)	356 ±16	426 ±33	19.7	0.0929
muscle wet weight (mg)				
gastrocnemius	1947 ±86	1539 ±28	-21.0†	0.0019
plantaris	327 ±12	341 ±8	4.3	0.3624
soleus	141 ±5	158 ±5	12.1†	0.0396
tibialis anterior	679 ±29	647 ±32	-4.7	0.4733
normalized muscle wet weight (mg/g bw)				
gastrocnemius	5.48 ±0.16	3.69 ±0.27	-32.7†	0.0005
plantaris	0.922 ±0.033	0.817 ±0.055	-11.4	0.1353
soleus	0.397 ±0.019	0.381 ±0.036	-4.0	0.6830
tibialis anterior	1.91 ±0.07	1.55 ±0.13	-18.8†	0.0432
myofiber CSA (µM^2)				
gastrocnemius	1493 ±83	822 ±29	-44.9†	0.0001
plantaris	641 ±49	681 ±31	6.2	0.5172
soleus	780 ±42	877 ±22	12.4	0.0744
**Metabolomic Study**	**15 months (n=8)**	**32 months (n=8)**	**% change**	***P*-value**
fasted body weight (g)	424 ±12	543 ±11	28.1†	0.000006

Values are expressed as means ± standard error; †, denotes significant differences (*P*-value < 0.05); *P*-value, *P*-value derived from a Student's 2-tailed t-Test
