# Supplementary material for: Metabolomic profiling reveals severe skeletal muscle group-specific perturbations of metabolism in aged FBN rats
Source: Biogerontology. 2014 Mar 21;15(3):217–32. doi: 10.1007/s10522-014-9492-5 (PMC4019835; doi:10.1007/s10522-014-9492-5)
Supplement: Supplementary file 4 — Supplementary material 4 (PDF 346 kb) [file 10522_2014_9492_MOESM4_ESM.pdf]

Metabolomic profiling reveals severe skeletal muscle group-specific perturbations of metabolism in aged FBN rats
S.M. Garvey[1] · J.E. Dugle[1] · A.D. Kennedy[2] · J.E. McDunn[2] · W. Kline[3] · L. Guo[2] · D.C. Guttridge[3] · S.L. Pereira[1] · N.K. Edens[1]
[1]Abbott Nutrition R&D, Columbus, OH · [2]Metabolon, Inc., Durham, NC · [3]The Ohio State University, Columbus, OH

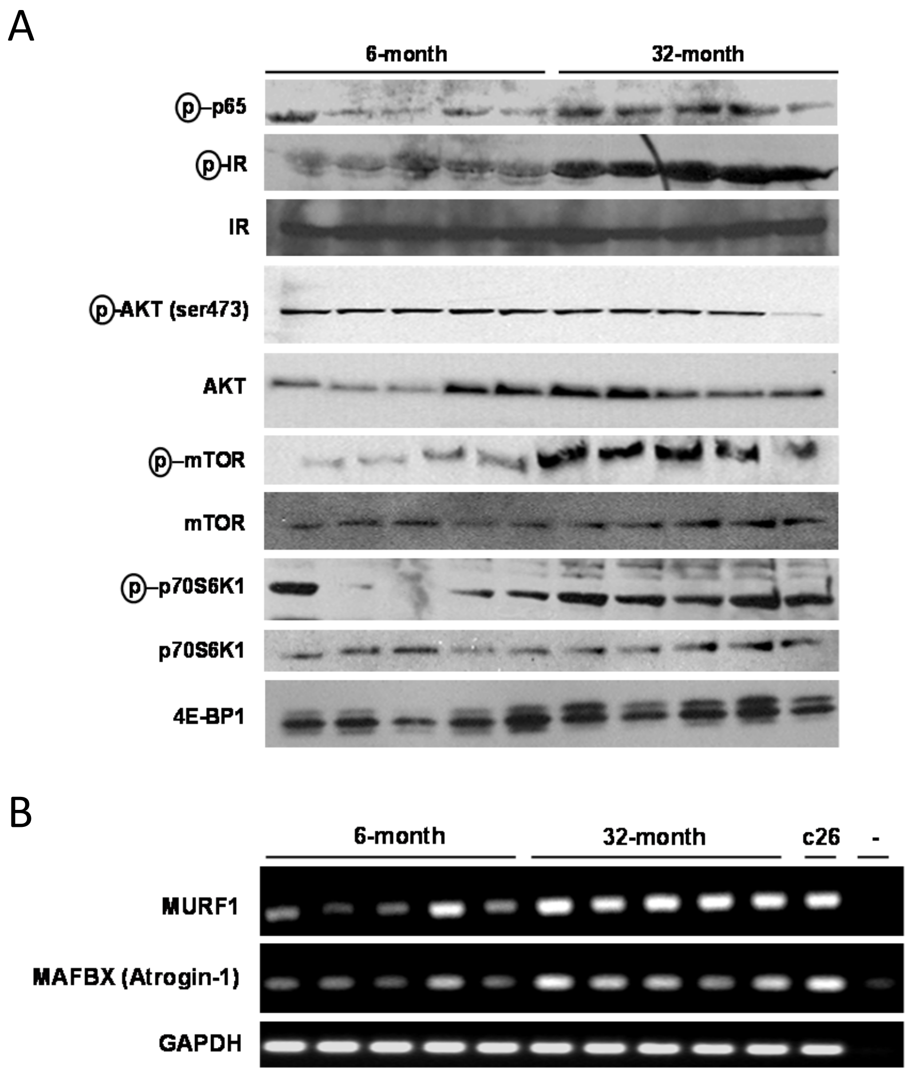

Online Resource 4 Molecular profiling of aged rat muscle. **a** Western blot analysis of gastrocnemius from 6- and 32-month-old FBN rats. **b** Semi-quantitative RT-PCR analysis of the protein degradation markers MURF1 and MAFBX (Atrogin-1) in the gastrocnemius muscles of 6-and 32-month-old rats. p65, transcription factor p65 or RELA or NFKB3; IR, insulin receptor; AKT, AKT1 or v-akt murine thymoma viral oncogene homolog 1; mTOR, mechanistic target of rapamycin; p70S6K1, ribosomal protein S6 kinase polypeptide 1; 4E-BP1, eukaryotic translation initiation factor 4E binding protein 1; MURF1, muscle RING-finger protein-1; MAFBX1, muscle atrophy F-box protein; GAPDH, glyceraldehyde-3-phosphate dehydrogenase; (p) , phospho; C26, positive control for MURF1/MAFBX upregulation from muscle of murine cancer cachexia model; "-", water control
