# Supplementary material for: Metabolomic profiling reveals severe skeletal muscle group-specific perturbations of metabolism in aged FBN rats
Source: Biogerontology. 2014 Mar 21;15(3):217–32. doi: 10.1007/s10522-014-9492-5 (PMC4019835; doi:10.1007/s10522-014-9492-5)
Supplement: Supplementary file 8 — Supplementary material 8 (PDF 38 kb) [file 10522_2014_9492_MOESM8_ESM.pdf]

Metabolomic profiling reveals severe skeletal muscle group-specific perturbations of metabolism in aged FBN rats

S.M. Garvey[1] · J.E. Dugle[1] · A.D. Kennedy[2] · J.E. McDunn[2] · W. Kline[3] · L. Guo[2] · D.C. Guttridge[3] · S.L. Pereira[1] · N.K. Edens[1]
[1]Abbott Nutrition R&D, Columbus, OH · [2]Metabolon, Inc., Durham, NC · [3]The Ohio State University, Columbus, OH

Online Resource 8 Alternate statistical analysis without imputation of metabolites that meet reporting threshold (i.e., metabolite detected in at least 6 of 8 samples) for each muscle. Heat map shows fold of change values between 32-month-old aged and 15-month-old adult groups within gastrocnemius and soleus datasets. Colored boxes represent statistically significant differences ($P < 0.05$). Red signifies increased levels in aged muscle. Green signifies decreased levels in aged muscle. RT, reporting threshold; M, months of age; DF, degrees of freedom; *P* , *P* -value Welch's two-sample t-test

Muscle Group	Biochemical	Meets RT (>70% fill) n (15M)	n (32M)	Log (means) 32M	15M	DF	*P* - value	Geometric means 32M	15M	FOLD OF CHANGE geometric means 32M:15M
gastroc	1-arachidonoylglycerophosphoethanolamine	8	8	5.0898	4.9485	13.7	0.2919	122980.8	88813.7	1.38
gastroc	1-arachidonoylglycerophosphoinositol	8	8	4.9680	4.9767	11.6	0.9524	92890.2	94781.4	0.98
gastroc	1-linoleoylglycerophosphoethanolamine	8	8	4.8361	4.8014	13.4	0.7546	68563.3	63294.1	1.08
gastroc	1-oleoylglycerophosphocholine	8	6	5.5321	5.3378	11.6	0.5261	340465.6	217649.4	1.56
gastroc	1-oleoylglycerophosphoethanolamine	8	8	5.1058	4.9596	11.2	0.5164	127576.9	91122.7	1.40
gastroc	1-palmitoylglycerol (1-monopalmitin)	8	8	5.5136	5.2741	9.1	0.1000	326256.1	187955.6	1.74
gastroc	1-palmitoylglycerophosphocholine	8	8	6.2322	6.2307	14.0	0.9963	1706978.7	1700979.2	1.00
gastroc	1-palmitoylglycerophosphoethanolamine	8	8	5.1879	5.0893	13.7	0.4020	154122.0	122827.6	1.25
gastroc	1-stearoylglycerophosphocholine	7	7	5.8746	5.8309	11.9	0.8685	749196.1	677446.0	1.11
gastroc	1-stearoylglycerophosphoethanolamine	6	6	5.5557	5.6603	9.4	0.5587	359489.8	457403.9	0.79
gastroc	1-stearoylglycerophosphoinositol	8	8	5.3657	5.3065	12.2	0.5680	232097.2	202530.8	1.15
gastroc	1,5-anhydroglucitol (1,5-AG)	8	8	5.6572	5.3680	9.7	0.0026	454141.7	233353.9	1.95
gastroc	1,6-anhydroglucose	8	7	5.2800	5.3201	12.3	0.6756	190528.0	208998.3	0.91
gastroc	10-heptadecenoate (17:1n7)	8	8	6.0751	6.0491	11.1	0.7693	1188650.1	1119788.9	1.06
gastroc	10-nonadecenoate (19:1n9)	8	8	5.7231	5.5796	11.8	0.1733	528534.7	379796.4	1.39
gastroc	2'-deoxycytidine	8	8	6.0073	5.7634	13.0	0.0000	1016895.8	579952.4	1.75
gastroc	2-aminobutyrate	8	8	5.4061	5.3192	11.6	0.1511	254725.4	208556.2	1.22
gastroc	2-arachidonoylglycerophosphocholine	7	7	5.8120	5.8303	11.2	0.9434	648594.1	676501.5	0.96
gastroc	2-arachidonoylglycerophosphoethanolamine	7	7	4.8689	4.7197	11.8	0.1769	73948.7	52446.0	1.41
gastroc	2-docosahexaenoylglycerophosphoethanolamine	7	6	6.1669	6.0952	10.8	0.8272	1468482.7	1245094.3	1.18
gastroc	2-docosapentaenoylglycerophosphoethanolamine	7	6	5.9980	5.8767	11.0	0.7396	995316.0	752841.1	1.32
gastroc	2-methylbutyroylcarnitine	8	8	5.4776	5.5102	13.1	0.4444	300316.1	323722.7	0.93
gastroc	2-oleoylglycerophosphocholine	7	6	5.2923	5.2321	9.8	0.8153	195998.8	170646.7	1.15
gastroc	2-oleoylglycerophosphoethanolamine	7	7	4.7590	4.7656	7.7	0.9804	57406.3	58291.9	0.98
gastroc	2-palmitoylglycerol (2-monopalmitin)	6	6	5.2112	5.2011	8.3	0.8667	162624.9	158885.2	1.02
gastroc	2-palmitoylglycerophosphocholine	6	6	5.6493	5.6034	9.9	0.8595	445945.0	401220.6	1.11
gastroc	2-palmitoylglycerophosphoethanolamine	6	6	5.5469	5.5763	10.0	0.9230	352323.7	376985.0	0.93
gastroc	3-(4-hydroxyphenyl)lactate	8	8	4.3803	4.3983	14.0	0.5954	24007.3	25021.1	0.96

gastroc	3-dehydrocarnitine	8	8	6.2430	6.4808	14.0	0.0000	1749942.7	3025192.7	**0.58**
gastroc	3-hydroxybutyrate (BHBA)	8	8	5.9529	5.8112	9.5	0.0541	897225.7	647387.0	1.39
gastroc	3-methyl-2-oxobutyrate	7	7	4.4131	4.2576	11.6	0.2962	25889.1	18098.4	1.43
gastroc	3-methyl-2-oxovalerate	8	8	4.3953	4.2877	13.5	0.3096	24848.5	19393.8	1.28
gastroc	3-methylhistidine	8	8	4.8058	4.6556	12.8	0.0073	63937.4	45250.4	**1.41**
gastroc	3-phosphoglycerate	8	8	6.6642	5.8458	7.8	0.0001	4615354.7	701094.3	**6.58**
gastroc	4-methyl-2-oxopentanoate	8	8	4.9987	4.7581	10.3	0.0971	99707.0	57298.1	1.74
gastroc	5-methylthioadenosine (MTA)	8	8	5.3552	5.2699	10.7	0.0324	226581.2	186155.7	**1.22**
gastroc	5-oxoproline	8	8	5.0874	4.8444	13.5	0.0023	122296.2	69894.0	**1.75**
gastroc	acetylcarnitine	8	8	7.6221	7.4937	7.7	0.1523	41886457.5	31170484.6	1.34
gastroc	adenosine 2'-monophosphate (2'-AMP)	8	8	4.4885	4.2498	13.3	0.0001	30795.7	17773.4	**1.73**
gastroc	adenosine 5'-monophosphate (AMP)	8	8	4.6999	4.9398	13.0	0.2329	50110.3	87061.0	0.58
gastroc	adrenate (22:4n6)	8	8	6.7026	6.5932	11.2	0.2596	5042250.6	3919009.7	1.29
gastroc	alanine	8	8	7.6881	7.6309	13.9	0.1524	48767600.5	42743599.1	1.14
gastroc	alanylleucine	8	8	5.6114	5.8961	8.9	0.0632	408656.8	787199.9	0.52
gastroc	alanyltyrosine	8	8	5.5237	6.1523	10.2	0.0002	333991.6	1419950.9	**0.24**
gastroc	alanylvaline	8	8	5.2851	5.6101	9.9	0.0082	192788.9	407484.6	**0.47**
gastroc	anserine	8	8	6.8508	6.9591	12.8	0.0000	7092436.0	9102197.3	**0.78**
gastroc	arachidonate (20:4n6)	8	8	6.9543	6.8563	12.6	0.2097	9001626.8	7183573.8	1.25
gastroc	arginine	8	8	6.4118	6.0486	12.1	0.0000	2580849.9	1118349.5	**2.31**
gastroc	asparagine	7	7	4.8979	4.6225	10.5	0.0296	79057.6	41932.4	**1.89**
gastroc	aspartate	8	8	6.2988	6.3604	12.2	0.5257	1989696.6	2293066.3	0.87
gastroc	aspartylphenylalanine	8	8	5.5384	5.3785	14.0	0.0394	345443.0	239035.4	**1.45**
gastroc	azelate (nonanedioate)	8	8	4.5810	4.5195	14.0	0.3005	38104.7	33078.7	1.15
gastroc	beta-alanine	8	8	5.6636	5.6151	11.6	0.5307	460921.9	412206.6	1.12
gastroc	betaine	8	8	6.5918	6.5110	13.4	0.0378	3906563.9	3243309.4	**1.20**
gastroc	butyrylcarnitine	8	8	6.4032	6.3497	12.7	0.4817	2530676.7	2237080.2	1.13
gastroc	C-glycosyltryptophan	8	8	5.3150	5.1495	13.7	0.0008	206519.2	141092.6	**1.46**
gastroc	campesterol	8	8	5.5472	5.3243	12.2	0.0087	352555.0	210991.8	**1.67**
gastroc	caproate (6:0)	8	8	4.8162	4.8741	12.5	0.4728	65495.8	74835.2	0.88
gastroc	carnitine	8	8	7.3906	7.5020	13.9	0.0002	24578520.5	31767999.7	**0.77**
gastroc	carnosine	8	8	6.1880	6.3644	13.8	0.0000	1541736.7	2314310.5	**0.67**
gastroc	cholate	8	8	5.0532	4.7035	9.4	0.0032	113034.7	50524.2	**2.24**
gastroc	cholesterol	8	8	6.7375	6.6142	10.3	0.3530	5464229.3	4112980.5	1.33
gastroc	choline	8	8	6.4753	6.1977	9.4	0.0053	2987183.4	1576405.5	**1.89**
gastroc	cis-vaccenate (18:1n7)	8	8	5.8956	5.4686	9.3	0.0023	786257.7	294193.1	**2.67**
gastroc	citrate	8	7	4.4102	4.7437	7.5	0.0128	25713.0	55423.7	**0.46**
gastroc	citrulline	8	8	6.1442	6.1346	9.5	0.7561	1393670.2	1363258.9	1.02

gastroc	creatine	8	8	6.7002	6.7276	13.0	0.0048	5014534.8	5340357.3	**0.94**
gastroc	creatinine	8	8	6.6098	6.6742	14.0	0.2103	4072112.3	4723329.2	0.86
gastroc	cysteine	8	8	5.6379	5.3970	14.0	0.0010	434370.0	249431.5	**1.74**
gastroc	cysteine-glutathione disulfide	8	8	5.9563	5.6393	13.8	0.0002	904173.9	435855.4	**2.07**
gastroc	cytidine	8	8	6.3981	5.9821	11.4	0.0000	2501171.8	959678.0	**2.61**
gastroc	cytidine 5'-monophosphate (5'-CMP)	8	7	5.1160	5.6105	8.6	0.0006	130612.8	407819.6	**0.32**
gastroc	dihomo-linoleate (20:2n6)	8	8	6.4398	6.3251	12.6	0.3151	2753054.7	2113832.1	1.30
gastroc	dihomo-linolenate (20:3n3 or n6)	8	8	6.0933	5.9062	12.4	0.0533	1239585.4	805834.4	1.54
gastroc	docosahexaenoate (DHA; 22:6n3)	8	8	6.1435	6.2261	12.4	0.3728	1391606.7	1682924.5	0.83
gastroc	docosapentaenoate (n3 DPA; 22:5n3)	8	8	6.5511	6.5011	10.4	0.5758	3556772.8	3170073.1	1.12
gastroc	eicosapentaenoate (EPA; 20:5n3)	8	8	5.9379	5.7926	13.9	0.1922	866788.4	620337.7	1.40
gastroc	eicosenoate (20:1n9 or 11)	8	8	6.4577	6.3887	12.2	0.5655	2869083.5	2447464.1	1.17
gastroc	ethanolamine	8	8	5.9230	5.8078	14.0	0.1411	837601.0	642449.5	1.30
gastroc	flavin adenine dinucleotide (FAD)	8	8	4.4645	4.4396	12.7	0.6709	29138.3	27517.8	1.06
gastroc	fructose	8	8	6.0131	5.9589	10.0	0.3210	1030675.6	909640.6	1.13
gastroc	fructose-6-phosphate	8	8	6.6609	6.6609	11.0	0.9995	4580629.4	4580070.6	1.00
gastroc	fumarate	8	7	5.5442	5.5690	9.4	0.7888	350091.1	370672.4	0.94
gastroc	galactitol (dulcitol)	8	7	4.7465	4.8834	11.8	0.1203	55785.6	76455.2	0.73
gastroc	glucose	8	8	7.2166	7.1422	12.5	0.0412	16464796.1	13874140.2	**1.19**
gastroc	glucose-6-phosphate (G6P)	8	8	7.1186	7.0788	10.0	0.6440	13141650.5	11990041.9	1.10
gastroc	glucose 1-phosphate	8	8	5.6187	5.5376	8.4	0.2986	415666.2	344832.4	1.21
gastroc	glutamate	8	8	6.7855	6.6554	12.7	0.0085	6102298.1	4522942.9	**1.35**
gastroc	glutamine	8	8	7.3766	7.2689	11.1	0.0033	23800506.9	18574345.4	**1.28**
gastroc	glutathione, oxidized (GSSG)	8	8	7.0016	6.9179	9.6	0.0247	10036547.7	8278185.1	**1.21**
gastroc	glutathione, reduced (GSH)	8	8	5.3973	5.2046	14.0	0.3426	249621.5	160173.3	1.56
gastroc	glycerate	8	8	5.7929	5.3747	12.7	0.0002	620761.2	236976.5	**2.62**
gastroc	glycerol	8	8	7.0887	7.0010	13.7	0.0169	12265621.0	10024037.6	**1.22**
gastroc	glycerol 2-phosphate	8	8	5.0113	4.8981	8.8	0.2940	102627.5	79083.1	1.30
gastroc	glycerol 3-phosphate (G3P)	8	8	6.4234	5.7506	14.0	0.0000	2650662.0	563137.9	**4.71**
gastroc	glycerophosphorylcholine (GPC)	8	8	6.2016	5.9590	13.8	0.0003	1590649.1	909818.9	**1.75**
gastroc	glycine	8	8	7.5026	7.5510	11.0	0.3883	31813659.9	35565522.1	0.89
gastroc	glycylisoleucine	7	7	4.9337	4.7847	11.8	0.0038	85844.0	60916.3	**1.41**
gastroc	glycylleucine	8	8	5.8015	5.6356	14.0	0.0002	633195.2	432161.3	**1.47**
gastroc	glycylproline	8	8	5.2201	5.0509	13.3	0.0296	166003.5	112445.6	**1.48**
gastroc	guanosine	8	8	4.8593	4.8689	13.8	0.9017	72334.6	73943.1	0.98
gastroc	guanosine 5'- monophosphate (GMP)	8	8	5.0575	5.1068	11.8	0.4774	114154.2	127870.5	0.89
gastroc	heptanoate (7:0)	8	8	4.4357	4.4631	11.9	0.7897	27273.2	29049.8	0.94
gastroc	hexanoylcarnitine	8	8	6.3272	6.1923	9.5	0.0810	2124420.5	1556870.2	1.36

gastroc	hippurate	8	8	4.3575	4.3568	12.6	0.9932	22777.5	22743.1	1.00
gastroc	histamine	8	8	6.3987	6.1502	7.5	0.0231	2504571.6	1413280.9	1.77
gastroc	histidine	8	8	4.9813	4.8132	10.6	0.0090	95783.8	65043.9	1.47
gastroc	hydroxyisovaleroyl carnitine	8	8	5.7687	5.8364	8.8	0.0247	587078.0	686156.6	0.86
gastroc	hypotaurine	8	8	5.9049	5.6238	12.1	0.0001	803328.0	420569.7	1.91
gastroc	hypoxanthine	8	8	5.9591	5.6773	8.9	0.0003	910058.3	475695.1	1.91
gastroc	inosine	8	8	6.8558	6.7959	10.6	0.0153	7175393.6	6250947.2	1.15
gastroc	Isobar: ribulose 5-phosphate, xylulose 5-phosphate	8	8	6.2582	5.7838	10.0	0.0011	1812021.1	607865.8	2.98
gastroc	isobutyrylcarnitine	8	8	5.4629	5.4638	12.6	0.9901	290310.3	290954.7	1.00
gastroc	isoleucine	8	8	7.5455	7.4233	8.4	0.0007	35118691.9	26504512.5	1.33
gastroc	isoleucylisoleucine	8	7	5.2883	5.4267	7.4	0.2696	194210.5	267146.4	0.73
gastroc	isoleucylleucine	8	8	5.5069	6.2664	9.2	0.0015	321280.8	1846536.9	0.17
gastroc	isovalerylcarnitine	8	8	5.3338	5.3296	14.0	0.9752	215667.6	213612.5	1.01
gastroc	kynurenate	7	8	4.4439	4.3681	10.5	0.5808	27790.2	23340.8	1.19
gastroc	kynurenine	8	8	5.8239	5.6963	11.3	0.3543	666585.1	496966.7	1.34
gastroc	lactate	8	8	8.4876	8.5971	14.0	0.0007	307318644.4	395495362.0	0.78
gastroc	laurate (12:0)	8	8	5.7922	5.8240	12.1	0.5386	619687.3	666869.3	0.93
gastroc	leucine	8	8	7.7187	7.5406	8.7	0.0001	52325012.4	34725075.8	1.51
gastroc	leucylleucine	8	7	5.0659	5.4715	11.7	0.0010	116373.5	296120.5	0.39
gastroc	linoleate (18:2n6)	8	8	7.6843	7.5925	11.2	0.2762	48340304.1	39127338.0	1.24
gastroc	linolenate [alpha or gamma; (18:3n3 or 6)]	8	8	6.6649	6.5492	13.1	0.3361	4622666.2	3541836.8	1.31
gastroc	lysine	8	8	6.6370	6.3586	8.1	0.0000	4335582.4	2283577.1	1.90
gastroc	malate	8	8	6.3760	6.2680	12.9	0.0776	2376953.9	1853450.1	1.28
gastroc	malonylcarnitine	8	8	6.1892	6.1571	13.6	0.6770	1546092.6	1435950.8	1.08
gastroc	maltose	8	8	6.4729	5.8230	14.0	0.0000	2971083.6	665333.7	4.47
gastroc	maltotetraose	8	8	5.5705	4.8081	14.0	0.0000	371972.9	64282.9	5.79
gastroc	maltotriose	8	8	6.6161	5.7655	14.0	0.0000	4131070.8	582807.3	7.09
gastroc	mannose	8	8	6.0331	5.6791	12.2	0.0035	1079169.6	477658.2	2.26
gastroc	mannose-6-phosphate	8	8	6.1988	6.1862	10.2	0.8802	1580352.6	1535280.3	1.03
gastroc	margarate (17:0)	8	8	6.2258	6.2097	11.9	0.8715	1681811.9	1620866.6	1.04
gastroc	methionine	8	8	6.9984	6.8679	11.8	0.0003	9964289.2	7376883.6	1.35
gastroc	myo-inositol	8	8	6.5459	6.3346	12.4	0.0191	3514644.4	2160638.4	1.63
gastroc	myristate (14:0)	8	8	6.7155	6.6811	11.0	0.6897	5194060.1	4797948.6	1.08
gastroc	myristoleate (14:1n5)	8	8	5.7675	5.6919	12.3	0.1579	585409.9	491950.9	1.19
gastroc	N-6-trimethyllysine	8	8	5.6876	5.3253	12.2	0.0002	487063.8	211487.6	2.30
gastroc	N-acetyl-aspartyl-glutamate (NAAG)	8	8	5.6283	5.4753	8.5	0.3339	424942.8	298768.0	1.42
gastroc	N-acetylaspartate (NAA)	8	8	5.1999	5.3523	10.8	0.3347	158445.9	225041.1	0.70
gastroc	N-acetylmethionine	8	8	4.8502	4.9204	11.4	0.2106	70834.8	83250.5	0.85

gastroc	N-acetylornithine	8	8	5.7919	5.8451	10.4	0.1007	619354.4	699953.2	0.88
gastroc	N-acetylthreonine	8	8	4.4056	4.4736	11.1	0.0972	25442.8	29757.2	0.86
gastroc	nicotinamide	8	8	7.4109	7.4306	12.1	0.3829	25759659.2	26955016.7	0.96
gastroc	nicotinamide adenine dinucleotide (NAD+)	8	8	4.4839	5.3542	10.0	0.0002	30474.1	226048.7	0.13
gastroc	octanoylcarnitine	8	8	5.6133	5.6204	12.2	0.9277	410528.5	417262.8	0.98
gastroc	oleate (18:1n9)	8	8	6.3479	5.9761	9.8	0.0018	2228160.0	946521.4	2.35
gastroc	ophthalmate	8	8	5.2632	5.1735	10.3	0.0809	183328.3	149100.8	1.23
gastroc	ornithine	8	8	5.1950	4.9718	13.9	0.0051	156691.8	93722.6	1.67
gastroc	palmitate (16:0)	8	8	7.6787	7.6217	12.5	0.3588	47720243.2	41854651.5	1.14
gastroc	palmitoleate (16:1n7)	8	8	7.2470	7.0315	12.5	0.0207	17658682.4	10752508.1	1.64
gastroc	palmitoylcarnitine	7	8	5.5093	5.8009	12.8	0.3113	323060.1	632262.1	0.51
gastroc	pantothenate	8	8	6.1791	6.2366	11.1	0.1933	1510579.2	1724068.5	0.88
gastroc	pentadecanoate (15:0)	6	6	5.2352	5.1588	7.7	0.2562	171866.6	144151.8	1.19
gastroc	phenol sulfate	8	8	4.2598	4.1659	10.6	0.2739	18189.6	14650.6	1.24
gastroc	phenylacetylglycine	8	8	4.0902	4.0592	10.4	0.7745	12308.7	11460.1	1.07
gastroc	phenylalanine	8	8	7.6618	7.5124	10.1	0.0001	45898262.3	32541352.6	1.41
gastroc	phosphate	8	8	8.5939	8.6591	14.0	0.0108	392539343.9	456190491.5	0.86
gastroc	phosphoethanolamine	6	7	5.2256	5.1808	7.6	0.6758	168113.2	151647.1	1.11
gastroc	proline	8	8	6.9619	6.8489	9.2	0.0010	9160936.1	7061838.4	1.30
gastroc	prolylleucine	8	8	6.1454	6.0170	13.8	0.0032	1397697.4	1039819.8	1.34
gastroc	propionylcarnitine	8	8	6.2639	6.4017	13.9	0.0002	1836292.3	2521695.6	0.73
gastroc	pseudouridine	7	6	4.8367	4.9347	9.6	0.1948	68662.4	86035.4	0.80
gastroc	pyroglutamine	8	8	5.3057	6.2434	11.4	0.0000	202150.8	1751350.4	0.12
gastroc	pyruvate	8	8	5.0083	4.8429	13.2	0.0059	101921.7	69648.5	1.46
gastroc	ribose	8	8	5.6866	5.3702	13.5	0.0006	485970.7	234515.1	2.07
gastroc	ribose 5-phosphate	8	8	5.7734	5.3281	11.2	0.0001	593501.7	212870.9	2.79
gastroc	ribulose	8	8	5.0148	4.5320	12.0	0.0002	103460.4	34040.9	3.04
gastroc	sarcosine (N-Methylglycine)	8	8	5.4696	5.5731	13.6	0.1478	294877.2	374209.1	0.79
gastroc	serine	8	8	7.0303	6.8042	12.3	0.0031	10721716.5	6370556.6	1.68
gastroc	sorbitol	8	8	5.2158	5.0108	13.9	0.0293	164348.5	102529.2	1.60
gastroc	spermidine	8	8	6.0734	5.8748	10.4	0.0359	1184011.2	749484.7	1.58
gastroc	sphingosine	6	6	5.3394	5.3366	8.7	0.9886	218468.4	217062.9	1.01
gastroc	stearate (18:0)	8	8	7.3122	7.2860	12.1	0.7285	20520260.1	19320544.3	1.06
gastroc	stearoyl sphingomyelin	8	8	6.1477	6.1281	13.9	0.6836	1404939.4	1343122.9	1.05
gastroc	succinylcarnitine	8	8	5.8379	6.0434	12.7	0.0002	688424.1	1105027.4	0.62
gastroc	taurine	8	8	6.3662	6.2556	14.0	0.0000	2323779.4	1801264.0	1.29
gastroc	threonine	8	8	6.3488	6.2702	13.6	0.0340	2232494.5	1862918.1	1.20
gastroc	trans-4-hydroxyproline	8	8	5.6533	5.7925	12.3	0.0392	450083.4	620167.0	0.73

gastroc	tryptophan	8	8	7.0561	6.9592	8.8	0.0073	11379882.1	9103188.3	1.25
gastroc	tyrosine	8	8	7.2365	7.1398	8.5	0.0016	17238244.2	13795937.6	1.25
gastroc	uracil	8	8	5.4624	5.0681	13.4	0.0008	289993.2	116970.3	2.48
gastroc	urea	8	8	6.7529	6.8525	13.9	0.0620	5661617.0	7120286.0	0.80
gastroc	uridine	8	8	5.5555	5.4651	12.5	0.0051	359343.1	291798.6	1.23
gastroc	urocanate	7	8	5.8227	5.4305	8.0	0.0423	664778.5	269471.7	2.47
gastroc	valerylcarnitine	8	8	5.1669	5.0575	13.8	0.1525	146858.7	114158.7	1.29
gastroc	valine	8	8	7.5121	7.3757	9.4	0.0003	32516679.4	23752194.0	1.37
gastroc	xanthine	8	8	6.7247	6.5553	9.6	0.0002	5305273.5	3591641.8	1.48
gastroc	xanthosine	6	6	4.8681	4.5944	7.8	0.0279	73803.8	39304.1	1.88
soleus	1-arachidonoylglycerophosphoethanolamine	8	8	4.9559	5.3126	12.6	0.0012	90337.9	205389.6	0.44
soleus	1-arachidonoylglycerophosphoinositol	8	8	4.9033	5.0748	11.7	0.0970	80034.9	118797.3	0.67
soleus	1-linoleoylglycerophosphoethanolamine	8	8	4.7708	4.9381	13.8	0.0819	58990.7	86725.8	0.68
soleus	1-oleoylglycerophosphoethanolamine	8	8	5.0183	5.0021	12.8	0.8622	104309.6	100496.1	1.04
soleus	1-palmitoylglycerol (1-monopalmitin)	8	8	4.9439	5.2272	11.1	0.0313	87890.9	168720.9	0.52
soleus	1-palmitoylglycerophosphocholine	8	8	5.5957	6.2465	14.0	0.0073	394217.6	1764101.1	0.22
soleus	1-palmitoylglycerophosphoethanolamine	8	8	5.0464	5.0650	13.8	0.8283	111287.9	116145.2	0.96
soleus	1-stearoylglycerophosphocholine	8	8	5.4122	6.1742	12.8	0.0056	258363.3	1493605.6	0.17
soleus	1-stearoylglycerophosphoethanolamine	8	7	5.1742	5.6195	13.0	0.0226	149352.2	416419.9	0.36
soleus	1-stearoylglycerophosphoinositol	8	8	5.4038	5.6689	14.0	0.0044	253409.3	466573.3	0.54
soleus	1,5-anhydroglucitol (1,5-AG)	8	8	5.4594	5.3663	9.6	0.3546	288006.8	232420.2	1.24
soleus	10-heptadecenoate (17:1n7)	8	8	6.0268	6.0608	14.0	0.4756	1063621.5	1150243.0	0.92
soleus	10-nonadecenoate (19:1n9)	8	8	5.6629	5.7448	14.0	0.1410	460178.1	555645.0	0.83
soleus	2'-deoxycytidine	8	8	5.9780	5.9557	9.5	0.4568	950696.8	903019.0	1.05
soleus	2-aminobutyrate	8	8	5.2291	5.3645	7.5	0.3456	169460.9	231471.4	0.73
soleus	2-arachidonoylglycerophosphoethanolamine	8	7	4.4962	4.9698	11.8	0.0023	31349.0	93289.4	0.34
soleus	2-docosahexaenoylglycerophosphoethanolamine	8	6	5.4548	6.0863	9.3	0.0261	284950.6	1219958.2	0.23
soleus	2-linoleoylglycerophosphocholine	8	6	5.2941	5.5950	6.1	0.0820	196832.5	393543.3	0.50
soleus	2-methylbutyroylcarnitine	8	8	5.6419	5.7229	13.1	0.1137	438404.5	528271.8	0.83
soleus	2-phosphoglycerate	7	7	4.6251	4.7277	11.4	0.5248	42175.7	53425.2	0.79
soleus	3-(4-hydroxyphenyl)lactate	8	8	4.0856	4.1425	8.0	0.1625	12180.1	13885.0	0.88
soleus	3-dehydrocarnitine	8	8	5.9641	6.2905	13.6	0.0000	920570.0	1952244.2	0.47
soleus	3-hydroxybutyrate (BHBA)	8	8	5.8548	5.7743	7.4	0.4359	715733.2	594752.2	1.20
soleus	3-indoxyl sulfate	6	7	3.8890	3.9084	9.9	0.8764	7743.8	8098.2	0.96
soleus	3-methyl-2-oxobutyrate	7	7	4.2894	4.2861	9.8	0.9823	19471.3	19325.9	1.01
soleus	3-methyl-2-oxovalerate	8	8	4.5043	4.4507	13.5	0.6673	31937.7	28227.6	1.13
soleus	3-methylhistidine	8	8	4.8897	4.8564	13.1	0.4700	77566.5	71843.9	1.08
soleus	3-phosphoglycerate	8	8	6.6987	6.6914	11.0	0.9458	4996737.7	4913239.5	1.02

soleus	4-hydroxybutyrate (GHB)	7	6	4.8142	4.8189	7.6	0.9356	65199.7	65908.0	0.99
soleus	4-methyl-2-oxopentanoate	8	8	4.9645	4.9245	13.0	0.7608	92152.4	84051.3	1.10
soleus	5-dodecenoate (12:1n7)	7	6	4.2550	4.2651	8.3	0.9129	17990.1	18413.6	0.98
soleus	5-methylthioadenosine (MTA)	8	8	5.1192	5.3061	13.5	0.0008	131585.5	202357.8	0.65
soleus	5-oxoproline	8	8	5.4818	5.4751	11.2	0.8340	303247.0	298606.2	1.02
soleus	acetylcarnitine	8	8	7.4246	7.5034	9.9	0.0240	26583991.0	31869753.3	0.83
soleus	adenosine 2'-monophosphate (2'-AMP)	8	8	4.3754	4.3546	13.9	0.7172	23734.5	22623.8	1.05
soleus	adenosine 3'-monophosphate (3'-AMP)	8	8	3.9619	4.0207	14.0	0.2206	9161.1	10487.1	0.87
soleus	adenosine 5'-diphosphate (ADP)	8	8	5.3912	5.7084	8.6	0.0031	246146.2	510995.7	0.48
soleus	adenosine 5'-monophosphate (AMP)	8	8	4.4656	4.5812	13.2	0.4299	29211.8	38124.8	0.77
soleus	adrenate (22:4n6)	8	8	6.6416	6.7519	14.0	0.1263	4381000.6	5648698.7	0.78
soleus	alanine	8	8	7.7030	7.9431	7.7	0.0072	50463182.1	87720583.7	0.58
soleus	anserine	8	8	6.6232	6.7321	8.5	0.0003	4199888.6	5395899.2	0.78
soleus	arachidonate (20:4n6)	8	8	7.0475	7.0406	13.0	0.9008	11156442.9	10979335.6	1.02
soleus	arginine	8	8	6.7486	6.5586	12.6	0.0000	5605523.6	3619198.2	1.55
soleus	ascorbate (Vitamin C)	7	6	5.3499	4.9084	7.0	0.1389	223811.5	80981.4	2.76
soleus	asparagine	8	7	5.4228	5.5212	11.1	0.2610	264717.2	332074.5	0.80
soleus	aspartate	8	8	7.0390	7.4392	7.6	0.0014	10938462.2	27492423.5	0.40
soleus	azelate (nonanedioate)	8	8	4.6472	4.5964	13.5	0.5850	44384.1	39482.4	1.12
soleus	beta-alanine	8	8	5.8519	5.9168	12.0	0.5133	711044.5	825608.9	0.86
soleus	beta-muricholate	6	7	3.9886	3.8827	9.0	0.2719	9741.0	7632.6	1.28
soleus	betaine	8	8	6.7947	6.7261	10.1	0.1422	6232408.9	5322285.6	1.17
soleus	butyrylcarnitine	8	8	6.1746	5.9013	11.3	0.0194	1494711.0	796680.0	1.88
soleus	C-glycosyltryptophan	8	8	5.5569	5.4588	12.6	0.0261	360519.3	287625.3	1.25
soleus	campesterol	8	7	5.5028	5.4506	12.5	0.3413	318281.4	282221.0	1.13
soleus	caproate (6:0)	8	8	4.8647	5.1593	14.0	0.0114	73236.0	144305.9	0.51
soleus	carnitine	8	8	7.1684	7.3110	8.4	0.0003	14736714.1	20465260.1	0.72
soleus	carnosine	8	8	6.2526	6.3269	12.2	0.0053	1788902.9	2122939.1	0.84
soleus	cholate	8	8	5.0152	4.8204	13.5	0.0083	103572.0	66128.3	1.57
soleus	cholesterol	8	8	6.4775	6.5758	9.1	0.3284	3002525.4	3765735.1	0.80
soleus	choline	8	8	6.9691	6.8594	11.8	0.0010	9313853.9	7234076.1	1.29
soleus	cis-vaccenate (18:1n7)	7	7	5.8137	5.7695	7.5	0.6185	651122.5	588230.6	1.11
soleus	citrulline	8	8	6.3335	6.3695	12.7	0.0874	2155286.8	2341762.0	0.92
soleus	creatine	8	8	6.5641	6.6249	12.0	0.0012	3665135.7	4216223.6	0.87
soleus	creatinine	8	8	6.2494	6.3824	13.7	0.0025	1775976.7	2412193.2	0.74
soleus	cysteine	8	8	5.6039	5.5397	12.4	0.6776	401653.2	346496.7	1.16
soleus	cysteine-glutathione disulfide	8	8	5.6461	5.4009	8.6	0.2593	442664.6	251706.9	1.76
soleus	cytidine	8	8	6.5117	6.5892	12.8	0.0415	3248414.9	3882909.4	0.84

soleus	cytidine 5'-monophosphate (5'-CMP)	8	8	4.9119	4.9775	13.2	0.0713	81635.6	94961.3	0.86
soleus	dihomo-linoleate (20:2n6)	8	8	6.3769	6.5329	13.8	0.0199	2381558.3	3411516.8	0.70
soleus	dihomo-linolenate (20:3n3 or n6)	8	8	6.1120	6.1215	12.8	0.8859	1294281.8	1322716.3	0.98
soleus	docosahexaenoate (DHA; 22:6n3)	8	8	6.1562	6.2735	13.5	0.1173	1432952.9	1877289.0	0.76
soleus	docosapentaenoate (n3 DPA; 22:5n3)	8	8	6.5040	6.6202	13.7	0.1416	3191288.3	4170216.9	0.77
soleus	eicosapentaenoate (EPA; 20:5n3)	8	8	6.0120	6.0572	13.9	0.4598	1027956.7	1140880.4	0.90
soleus	eicosenoate (20:1n9 or 11)	8	8	6.3505	6.5914	14.0	0.0011	2241475.0	3903420.8	0.57
soleus	ethanolamine	8	8	5.6909	5.8996	12.3	0.0803	490770.1	793549.4	0.62
soleus	flavin adenine dinucleotide (FAD)	8	8	4.4679	4.6922	9.8	0.0002	29367.2	49230.1	0.60
soleus	fructose	8	8	5.6791	5.9514	8.6	0.0061	477658.8	894054.6	0.53
soleus	fructose-6-phosphate	8	8	6.2575	6.5114	8.7	0.0226	1809377.8	3246313.3	0.56
soleus	fumarate	8	8	5.5839	5.8446	13.4	0.0141	383582.6	699151.6	0.55
soleus	gamma-glutamylleucine	8	8	5.1430	5.2322	10.5	0.0073	139007.4	170687.0	0.81
soleus	glucose	8	8	7.0356	7.2136	8.3	0.0325	10855433.1	16351884.9	0.66
soleus	glucose-6-phosphate (G6P)	8	8	6.7175	6.9770	9.4	0.0137	5217401.6	9484472.4	0.55
soleus	glucose 1-phosphate	8	8	5.4108	5.6115	9.1	0.0500	257537.6	408748.3	0.63
soleus	glutamate	8	8	7.0369	7.0895	10.8	0.0104	10886283.4	12288875.6	0.89
soleus	glutamine	8	8	7.6355	7.6335	13.4	0.8754	43204040.4	43001578.9	1.00
soleus	glutathione, oxidized (GSSG)	8	8	7.2037	7.3113	13.2	0.0002	15984092.8	20477950.2	0.78
soleus	glutathione, reduced (GSH)	8	8	6.4536	6.7588	9.3	0.3369	2842061.8	5738311.4	0.50
soleus	glycerate	8	8	5.2263	5.4154	8.8	0.0771	168364.8	260250.9	0.65
soleus	glycerol	8	8	7.0040	7.0719	7.7	0.3013	10092727.5	11801732.8	0.86
soleus	glycerol 2-phosphate	7	8	4.7412	5.0273	11.0	0.0041	55112.3	106493.5	0.52
soleus	glycerol 3-phosphate (G3P)	8	8	6.5140	6.7039	9.0	0.0943	3266073.2	5057114.4	0.65
soleus	glycerophosphorylcholine (GPC)	8	8	6.3657	6.4372	12.5	0.2044	2321278.6	2736404.8	0.85
soleus	glycine	8	8	7.2281	7.3326	7.8	0.2442	16909518.8	21505937.0	0.79
soleus	glycylisoleucine	8	7	5.0415	5.0731	12.0	0.5743	110037.0	118338.3	0.93
soleus	glycylleucine	8	8	5.7149	5.7473	12.1	0.5553	518651.5	558838.1	0.93
soleus	glycylproline	6	8	4.9970	4.9856	11.8	0.8681	99321.7	96732.2	1.03
soleus	guanosine	8	8	3.8270	4.3470	12.1	0.0000	6714.4	22233.6	0.30
soleus	guanosine 5'- monophosphate (GMP)	8	7	4.8875	4.9348	12.2	0.5017	77171.5	86054.3	0.90
soleus	heptanoate (7:0)	7	7	4.2875	4.3204	11.8	0.6548	19386.0	20913.4	0.93
soleus	hexanoylcarnitine	8	8	6.0469	5.9135	9.9	0.3419	1114081.4	819389.8	1.36
soleus	hippurate	8	8	4.4330	4.5397	13.2	0.4322	27098.8	34649.9	0.78
soleus	histamine	8	8	6.4013	6.3813	10.1	0.6743	2519237.4	2405824.7	1.05
soleus	histidine	8	8	5.4143	5.4162	7.8	0.9539	259622.3	260754.7	1.00
soleus	hydroxyisovaleroyl carnitine	8	8	5.5399	5.8625	10.3	0.0000	346683.2	728593.9	0.48
soleus	hypotaurine	8	8	6.0563	6.2660	10.0	0.0570	1138400.5	1845002.3	0.62

soleus	hypoxanthine	8	8	5.9767	6.0733	8.4	0.0066	947784.7	1183786.4	0.80
soleus	inosine	8	8	6.4802	6.7184	10.0	0.0000	3021597.0	5229173.8	0.58
soleus	Isobar: fructose 1,6-diphosphate, glucose 1,6-diphosphate	6	7	4.2361	4.1293	10.4	0.3594	17222.7	13466.5	1.28
soleus	Isobar: ribulose 5-phosphate, xylulose 5-phosphate	8	8	6.1396	6.3933	8.8	0.0129	1379089.7	2473581.7	0.56
soleus	isobutyrylcarnitine	8	8	5.5820	5.8915	11.1	0.0009	381983.5	778987.5	0.49
soleus	isoleucine	8	8	7.7154	7.6439	13.9	0.0786	51927474.1	44041562.5	1.18
soleus	isovalerylcarnitine	8	8	5.2558	5.3153	11.4	0.5282	180221.1	206699.4	0.87
soleus	kynurenate	8	8	4.5687	4.4142	13.8	0.2999	37044.1	25953.6	1.43
soleus	kynurenine	8	8	5.9500	5.7145	13.8	0.1347	891228.7	518258.8	1.72
soleus	lactate	8	8	7.8048	8.1201	7.5	0.0028	63803431.5	131852247.1	0.48
soleus	laurate (12:0)	8	8	5.7580	5.7970	13.9	0.2768	572841.4	626680.9	0.91
soleus	leucine	8	8	7.9510	7.8321	13.8	0.0117	89323568.4	67933115.1	1.31
soleus	linoleate (18:2n6)	8	8	7.6556	7.7212	13.8	0.0415	45251939.8	52631212.6	0.86
soleus	linolenate [alpha or gamma; (18:3n3 or 6)]	8	8	6.6959	6.8108	14.0	0.0232	4964682.3	6468883.7	0.77
soleus	lysine	8	8	6.8147	6.6894	9.0	0.0006	6527487.8	4891109.8	1.33
soleus	malate	8	8	6.3647	6.7414	7.5	0.0016	2315691.2	5513781.0	0.42
soleus	malonylcarnitine	8	8	6.0492	6.0698	14.0	0.8019	1120065.9	1174438.8	0.95
soleus	maltohexaose	8	8	4.8685	4.9305	12.2	0.3638	73879.4	85217.9	0.87
soleus	maltopentaose	8	8	6.0302	6.0691	13.4	0.5333	1072048.7	1172590.0	0.91
soleus	maltose	8	8	6.6283	6.7240	12.8	0.2961	4249030.2	5296988.0	0.80
soleus	maltotetraose	8	8	5.9404	6.0143	13.5	0.2729	871774.3	1033437.9	0.84
soleus	maltotriose	8	8	6.6998	6.9207	11.2	0.0446	5009631.9	8330329.6	0.60
soleus	mannose	8	8	5.7869	5.8713	11.6	0.5226	612249.3	743613.6	0.82
soleus	mannose-6-phosphate	8	8	5.7427	5.9665	10.8	0.0113	552917.1	925673.7	0.60
soleus	margarate (17:0)	8	8	6.0661	6.2963	14.0	0.0073	1164296.9	1978561.0	0.59
soleus	methionine	8	8	7.2669	7.1678	13.7	0.0302	18487415.6	14716862.6	1.26
soleus	myo-inositol	8	8	6.6184	6.5888	7.4	0.7325	4153340.8	3880029.8	1.07
soleus	myristate (14:0)	8	8	6.6466	6.7012	13.8	0.2694	4431829.7	5025311.6	0.88
soleus	myristoleate (14:1n5)	8	8	5.7925	5.7930	11.8	0.9896	620135.7	620937.3	1.00
soleus	N-6-trimethyllysine	8	8	6.0374	5.9249	12.5	0.1477	1090046.3	841153.2	1.30
soleus	N-acetyl-aspartyl-glutamate (NAAG)	8	8	5.5443	5.5507	12.7	0.9059	350177.8	355403.2	0.99
soleus	N-acetylaspartate (NAA)	8	7	4.8630	4.9448	11.1	0.4198	72942.5	88067.7	0.83
soleus	N-acetylmethionine	8	8	4.9887	4.8913	11.9	0.0662	97438.0	77852.7	1.25
soleus	N-acetylornithine	8	8	5.6923	5.8115	11.0	0.0075	492417.2	647900.8	0.76
soleus	N-acetylthreonine	8	8	4.2606	4.2881	10.6	0.4804	18223.6	19413.6	0.94
soleus	nicotinamide	8	8	7.2146	7.3579	8.1	0.0007	16392054.7	22796848.3	0.72
soleus	octanoylcarnitine	6	6	5.0528	5.1416	9.3	0.4992	112921.1	138536.0	0.82

soleus	oleate (18:1n9)	8	8	6.2785	6.2921	9.3	0.8787	1898836.4	1959174.2	0.97
soleus	ophthalmate	8	8	5.4146	5.4804	8.3	0.3762	259787.8	302239.8	0.86
soleus	ornithine	8	8	5.3066	5.3959	11.0	0.4105	202563.2	248800.5	0.81
soleus	palmitate (16:0)	8	8	7.6304	7.6686	14.0	0.2225	42698875.7	46628044.5	0.92
soleus	palmitoleate (16:1n7)	8	8	7.2318	7.1573	13.2	0.1610	17051321.8	14365744.5	1.19
soleus	pantothenate	8	8	6.4577	6.7300	12.8	0.0000	2868896.2	5370877.1	0.53
soleus	pentadecanoate (15:0)	7	7	5.0470	5.1569	7.9	0.1530	111419.7	143502.3	0.78
soleus	phenol sulfate	8	8	4.2196	4.2111	11.1	0.9461	16580.4	16260.9	1.02
soleus	phenylacetylglycine	8	8	4.1857	4.3899	14.0	0.1894	15334.4	24543.6	0.62
soleus	phenylalanine	8	8	7.8778	7.7892	13.6	0.0404	75467650.7	61547039.7	1.23
soleus	phosphate	8	8	8.3239	8.5201	7.2	0.0175	210816234.4	331234373.8	0.64
soleus	phosphoenolpyruvate (PEP)	8	7	5.6281	5.7566	6.8	0.5779	424723.8	570916.3	0.74
soleus	phosphoethanolamine	8	8	5.2094	5.4938	9.4	0.1109	161968.9	311719.9	0.52
soleus	phosphopantetheine	7	6	5.0017	5.0157	10.4	0.9013	100402.2	103683.0	0.97
soleus	proline	8	8	7.2894	7.1802	13.1	0.0062	19470806.2	15141519.9	1.29
soleus	prolylleucine	8	8	5.9920	6.0038	12.3	0.8326	981795.5	1008725.0	0.97
soleus	propionylcarnitine	8	8	5.9999	6.2527	13.8	0.0000	999730.6	1789405.8	0.56
soleus	pyroglutamine	8	8	5.5726	6.6067	10.4	0.0000	373801.0	4042867.4	0.09
soleus	pyruvate	7	8	4.6093	4.7516	12.6	0.0341	40676.0	56443.1	0.72
soleus	ribose	8	8	5.5820	5.7495	8.6	0.0416	381913.1	561708.4	0.68
soleus	ribose 5-phosphate	8	8	5.5752	5.6780	13.7	0.4371	375985.5	476382.9	0.79
soleus	ribulose	8	8	4.9624	5.1881	10.0	0.0141	91699.2	154203.4	0.59
soleus	sarcosine (N-Methylglycine)	8	7	5.1709	5.3103	7.1	0.0752	148226.4	204328.9	0.73
soleus	sedoheptulose-7-phosphate	8	8	5.0090	5.1158	11.7	0.2989	102084.4	130563.8	0.78
soleus	serine	8	8	7.3808	7.4533	7.5	0.3297	24033293.6	28399686.8	0.85
soleus	sorbitol	8	8	5.1980	5.3454	9.9	0.2872	157749.9	221517.7	0.71
soleus	spermidine	8	8	6.2152	5.9543	11.4	0.0035	1641171.7	900134.3	1.82
soleus	stearate (18:0)	8	8	7.2422	7.3697	13.8	0.0174	17467514.2	23426561.5	0.75
soleus	stearoyl sphingomyelin	8	7	5.5606	5.6707	6.5	0.3689	363550.3	468535.8	0.78
soleus	succinylcarnitine	8	8	5.9857	6.3232	8.2	0.0001	967575.8	2104887.6	0.46
soleus	taurine	8	8	6.2893	6.3144	14.0	0.1732	1946772.4	2062298.2	0.94
soleus	threonine	8	8	6.5254	6.4235	12.3	0.0043	3352647.2	2651581.3	1.26
soleus	trans-4-hydroxyproline	8	7	5.4897	5.6677	8.5	0.2515	308831.9	465296.9	0.66
soleus	tryptophan	8	8	7.2258	7.1147	14.0	0.0183	16819080.7	13024012.2	1.29
soleus	tyrosine	8	8	7.4875	7.3596	14.0	0.0041	30727251.4	22887438.4	1.34
soleus	uracil	8	8	5.7527	5.8763	8.6	0.1528	565879.1	752136.4	0.75
soleus	urea	8	8	6.4047	6.8427	7.2	0.0495	2539440.4	6962048.4	0.36
soleus	uridine	8	8	4.9518	5.3740	14.0	0.0000	89498.0	236589.5	0.38

soleus	urocanate	8	8	6.0207	5.6223	12.8	0.0043	1048774.4	419047.5	**2.50**
soleus	valerylcarnitine	6	8	5.1065	5.0822	9.4	0.6781	127789.9	120836.1	1.06
soleus	valine	8	8	7.6674	7.5927	14.0	0.0924	46492425.8	39144812.9	1.19
soleus	xanthine	8	8	6.7497	6.7676	12.5	0.4084	5620066.6	5855786.0	0.96
soleus	xanthosine	8	8	5.1652	5.2225	12.9	0.4863	146277.1	166912.3	0.88
soleus	xylulose	7	7	4.7513	4.9092	11.8	0.0750	56402.4	81129.2	0.70
