# Supplementary material for: Metabolomic profiling reveals severe skeletal muscle group-specific perturbations of metabolism in aged FBN rats
Source: Biogerontology. 2014 Mar 21;15(3):217–32. doi: 10.1007/s10522-014-9492-5 (PMC4019835; doi:10.1007/s10522-014-9492-5)
Supplement: Supplementary file 9 — Supplementary material 9 (PDF 100 kb) [file 10522_2014_9492_MOESM9_ESM.pdf]

S.M. Garvey[1] · J.E. Dugle[1] · A.D. Kennedy[2] · J.E. McDunn[2] · W. Kline[3] · L. Guo[2] · D.C. Guttridge[3] · S.L. Pereira[1] · N.K. Edens[1]
[1]Abbott Nutrition R&D, Columbus, OH · [2]Metabolon, Inc., Durham, NC · [3]The Ohio State University, Columbus, OH

Online Resource 9 Metabolites that are significantly different with aging in _both_ gastrocnemius and soleus muscles. Heat map shows fold of change values between 32-month-old aged and 15-month-old adult groups within gastrocnemius and soleus datasets. Colored boxes represent statistically significant differences ($P < 0.05$). Red signifies increased levels in aged muscle. Green signifies decreased levels in aged muscle.

Biochemical	Fold of Change	
	Gastroc 32M:15M	Soleus 32M:15M
glucose ●	1.19	0.71
lactate ●	0.78	0.52
maltotriose ●	7.06	0.64
ribose ●	2.04	0.73
ribulose ●	2.88	0.63
ribulose 5-phosphate + xylulose 5-phosphate ●	2.61	0.60
succinylcarnitine ●	0.63	0.47
anserine ●	0.78	0.78
arginine ●	2.27	1.55
C-glycosyltryptophan ●	1.46	1.27
carnitine ●	0.77	0.73
carnosine ●	0.67	0.85
creatine ●	0.94	0.87
hydroxyisovaleroyl carnitine ●	0.86	0.48
glutamate ●	1.34	0.89
leucine ●	1.52	1.32
lysine ●	1.92	1.32
methionine ●	1.36	1.25
phenylalanine ●	1.42	1.22
proline ●	1.31	1.29
pyroglutamine ●	0.13	0.10
spermidine ●	1.47	1.91
threonine ●	1.19	1.26
tryptophan ●	1.26	1.29
tyrosine ●	1.26	1.34
urocanate ●	2.28	2.69
3-dehydrocarnitine ●	0.58	0.47
cholate ●	2.01	1.54
choline ●	1.73	1.28
propionylcarnitine ●	0.73	0.56
5-methylthioadenosine (MTA) ●	1.23	0.65
cytidine ●	2.67	0.84
hypoxanthine ●	1.84	0.81
inosine ●	1.14	0.58
uridine ●	1.23	0.38
glutathione, oxidized (GSSG)	1.23	0.78
phosphate	0.86	0.67

● carbohydrate ● TCA cycle ● lipid ● amino acid ● nucleotide
